# Tumor microenvironment signatures enhances lung adenocarcinoma prognosis prediction: Implication of intratumoral microbiota

**DOI:** 10.15698/mic2025.08.855

**Published:** 2025-08-11

**Authors:** Fei Zhao, Lei Wang, Dongjie Du, Heaven Zhao, Geng Tian, Yufeng Li, Yankun Liu, Zhiwu Wang, Dasheng Liu, Jingwu Li, Lei Ji, Hong Zhao

**Affiliations:** 1 School of Mathematical Sciences, Ocean University of China, Qingdao, 266100, China.; 2 Hebei Key Laboratory of Molecular Oncology, Hebei, 063000, China.; 3 Tangshan Key Laboratory of Cancer Prevention and Therapy, Hebei, 063000, China.; 4 Department of Pathology, Tangshan People's Hospital, Hebei, 063000, China.; 5 Department of Vascular Surgery, Hebei General Hospital, Shijiazhuang, 050051, China.; 6 Geneis Beijing Co., Ltd., Beijing, 100102, China.; 7 Qingdao Geneis Institute of Big Data Mining and Precision Medicine, Qingdao 266000, China.; 8 The Cancer Institute, Tangshan People's Hospital, Hebei, 063000, China.; 9 Tangshan Key Laboratory of Precision Medicine Testing, Hebei, 063000, China.; 10 Department of Chemoradiotherapy, Tangshan People's Hospital, Hebei, 063000, China.; 11 Department of Thoracic Surgery, Jiamusi Central Hospital, Jiamusi, 154000, China.; 12 Department of Gastrointestinal Surgery, Tangshan People's Hospital, Hebei, 063000, China.; a The authors contributed equally to this work.

**Keywords:** intratumoral microbiome, lung adenocarcinoma, machine learning, prognosis, tumor microenvironment

## Abstract

The interaction between intratumoral microbiome and the tumor microenvironment (TME) has furthered our understanding of tumor ecology. Yet, the implications of their interaction for lung cancer management remain unclear. In the current work, we collected host transcriptome samples and matched intratumoral microbiome samples, as well as detailed clinical metadata from The Cancer Genome Atlas (TCGA) of 478 patients with lung adenocarcinoma (LUAD). Utilizing the multiomics integration approach, we comprehensively investigated the crosstalk between the TME and intratumoral microbiome in patients with LUAD. First, we developed a prognostic model based on the TME signatures (TMEindex) that clearly distinguished clinical, survival, and response to immunotherapy of patients with LUAD. Additionally, we found profound differences in intratumoral microbiota signatures, including alpha- and beta-diversity, among patients with different survival risks based on the TME signatures. In depth, we detected that genera *Luteibacter *and *Chryseobacterium *were strongly negatively and positively associated with patients’ survival risk, respectively, suggesting their opposing roles in cancer progression. Moreover, we developed a model that fused intratumoral microbial abundance information with TME signatures, called intratumoral microbiome-modified TMEindex (IMTMEindex), leading in predicting patient overall survival at 1-, 3-, and 5-years. Future clinical profiling of the specific intratumoral microbes in the TME could improve prognosis, inform immunotherapy, and facilitate the development of novel therapeutics for LUAD.

## Abbreviations

DEG - differentially expressed gene,

ICGs - immune checkpoint-related genes,

ICIs - immune checkpoint inhibitors,

LUAD - lung adenocarcinoma,

NK - natural killer,

NSCLC - non-small cell lung cancer,

OS - overall survival,

TCGA - The Cancer Genome Atlas,

TIDE - Tumor immun dysfunction and exclusion,

TME - tumor microenvironment,

WGCNA - Weighted Gene Co-expression Network Analysis.

## INTRODUCTION

Lung cancer remains the leading cause of cancer-related death worldwide 1,2. Lung adenocarcinoma (LUAD) is the most common type of lung cancer, belonging to the non-small cell cancer, accounting for 30% to 35% of all lung cancers 3. Despite advances in treatment strategies, five-year survival for patients with LUAD remains limited 4. At present, the tumor-related mechanism of LUAD remains unclear 5, and the heterogeneity of tumors makes it difficult to accurately assess the prognosis of each patient 6. Therefore, accurate and individualized assessment and improvement of survival in patients with LUAD remains a huge challenge.

The tumor microenvironment (TME) is the environment in which the tumor resides and is composed of various immune cells, stromal cells (including mesenchymal cells, endothelial cells), extracellular matrix molecules, and a variety of cytokines. Factors such as immune cells, angiogenesis, and cytokines in the TME are closely related to the biological characteristics of the tumor, and the components in the TME can define the immunophenotype of cancer, thus affecting the development of the tumor and the prognosis of patients 7,8. Various cells in TME can be tumor suppressor or tumor supportive 9. For example, low levels of infiltration of cytotoxic immune cells can help tumor cells evade immune attack, thereby reducing a patient's probability of survival. In addition to immune cells, stromal cells also regulate tumor immunophenotypes, such as cancer-associated fibroblasts, which exert a direct immunosuppressive mechanism of action 10. In addition, abnormal changes in the TME not only affect patient prognosis, but can also be used as a biomarker for treatment, such as immunotherapy. Finding characteristic markers from immune cells and stromal cells may be an effective way to predict the prognosis and benefit of treatment.

Tumor tissues once thought to be sterile are actually home to a variety of microorganisms, which have been shown to be closely related to tumorigenesis 11,12,13,14. Microorganisms in tumors can significantly change the biological characteristics of different cell compartments and affect the occurrence, development and metastasis of tumors as well as anti-tumor immunity 15,16,17,18. Anti-tumor immune activity can be stimulated or inhibited by signaling pathways, which in turn can consist of polysaccharides of microbial origin. This association underscores the importance that changes in the microbiome may lead to a more or less favorable tumor response 19,20. The analysis of the intratumoral microbiome associated with lung cancer has only just begun and some revelatory studies have been conducted. For example, one study suggested that the number of *Veillonella* and *Megasphaera *in patients with lung cancer was significantly higher than that in patients with benign disease 21. Yu *et al*. reported that the microbiota of lung tumor tissues showed significantly lower alpha diversity compared to non-malignant lung tissue samples, and that bacterial composition was correlated with cancer stage 22.

Studies have shown that intratumoral microbiota affect tumor progression mainly by changing the tumor immune microenvironment and regulating local immune response 23,24,25,26,27. Reducing the number of bacteria in the lungs of mice by antibiotic aerosol inhalation can activate tumor-infiltrating T cells and natural killer (NK) cells, reduce the number of immunosuppressive regulatory T cells, and enhance local anti-tumor immune response 28,29. Increased local bacterial load and changes in microbial composition can stimulate myeloid immune cells to produce IL-1β and IL-23 through the myD88-dependent pathway, and these cytokines stimulate the activation and proliferation of Vγ6^+^ Vδ1^+^ γδ T cells, and then produce IL-17 and other effector molecules to promote inflammation and tumourgenesis 30. The intratumoral microbiota can modulate anti-tumor immunity through its interaction with the TME and influence the efficacy of cancer immunotherapy represented by immune checkpoint inhibitors (ICIs).

It has been widely demonstrated that TME signatures and intratumoral microbial profiles are independently associated with lung tumors. However, previous studies have neither systematically established a connection between the two nor considered their combined prognostic effects. To bridge this gap, this study incorporated a total of 478 patients with LUAD from The Cancer Genome Atlas (TCGA). Through a systematic and integrated analysis of multiple cell populations and microbiota within the TME, as well as clinical phenotypes and prognostic outcomes, we discovered that the abundance of specific intratumoral microbes was strongly correlated with the survival risk predicted by a model constructed based on TME characteristics. This finding suggests the existence of crosstalk between intratumoral microbes and the TME, and highlights its impact on patient prognosis. Our study offers a valuable opportunity to identify microbial-based biomarkers for cancer prevention or prognosis. This, in turn, can aid in the design and testing of therapeutic strategies aimed at modulating the microbiota to improve cancer treatment and, ultimately, even prevent or intercept malignant transformation.

## RESULTS

### Identification of TME-related genes

**Figure 1:**
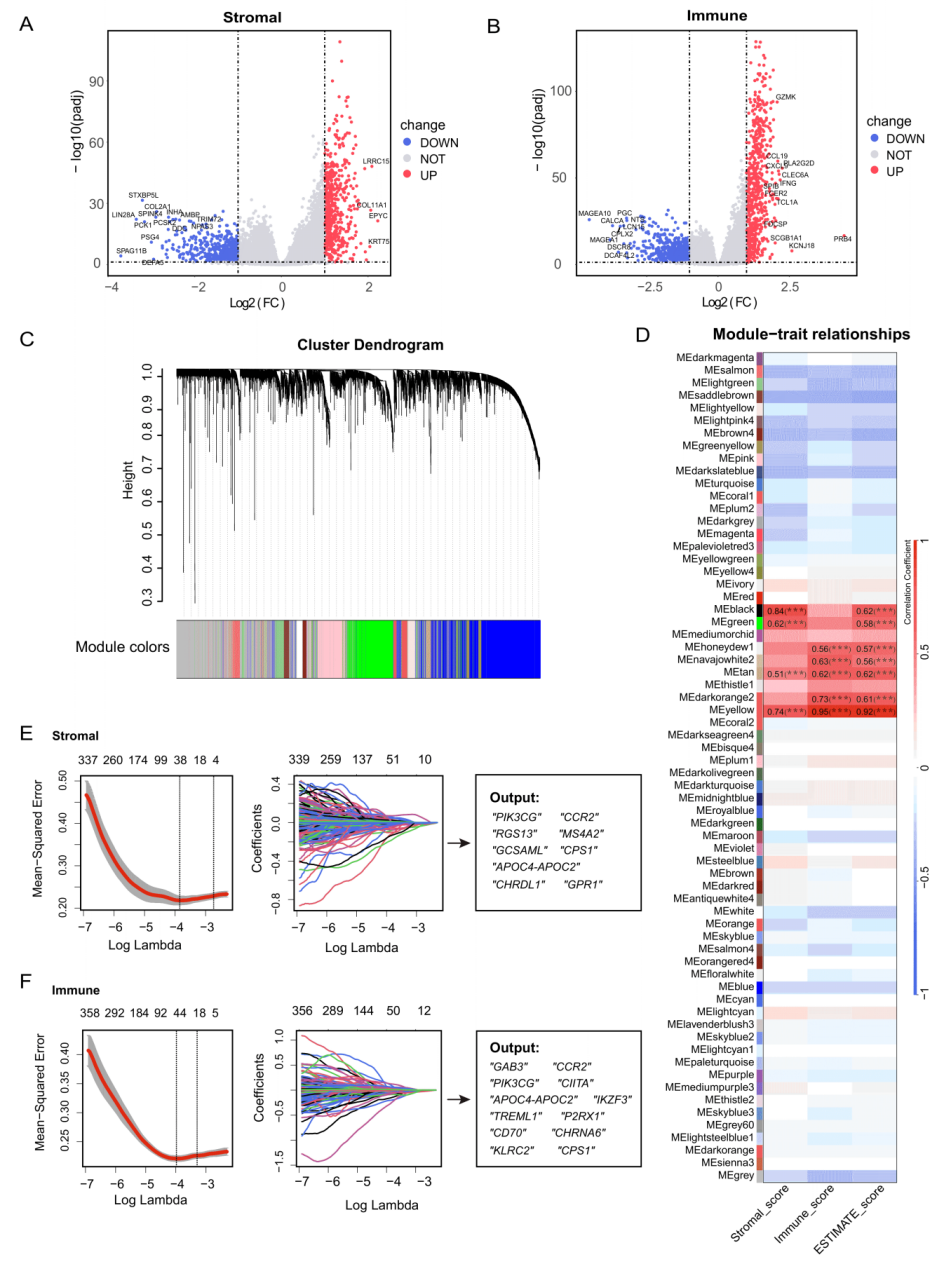
FIGURE 1: Identification of the TME-related genes. **(A)** Differentially expressed genes between patients with high and low stromal scores. **(B)** Differentially expressed genes between patients with high and low immune scores. **(C)** Gene modules recognized by WGCNA. **(D)** Correlation between gene modules and stromal score, immune score and ESTIMATE score. **(E-F)** LASSO algorithm was used to further screen genes extracted from WGCNA. ***P < 0.001.

First, the Estimation of STromal and Immune cells in Malignant Tumor tissues using Expression data (ESTIMATE) algorithm was used to obtain the stromal and immune scores of all patients. In order to define TME-related genes, differentially expressed gene (DEG) analysis was carried out on the two groups with different stromal and immune scores (**Fig. 1A** and **1B**). Weighted Gene Co-expression Network Analysis (WGCNA) delimited distinguishable modules by hierarchical clustering (**Fig. 1C**), and the results showed that the black, green, brown, and yellow modules were strongly positively correlated with stromal score, while the honeydew1, navajowhite2, brown, darkorange2, and yellow modules were strongly positively correlated with immune score (**Fig. 1D**).

Next, the intersection of the identified DEGs with genes in the WGCNA modules was used as input to least absolute shrinkage and selection operator (LASSO)-Cox regression from the perspective of stromal and immune scores, respectively. From the stromal perspective, *PIK3CG*, *CCR2*, *RGS13*, *MS4A2*, *GCSAML*, *APOC4-APOC2*, *CHRDL1*, *GPR1*, and *CPS1* genes were identified; meanwhile, from the immune perspective, *GAB3*, *CCR2*, *PIK3CG*, *CIITA*, *APOC4-APOC2*, *IKZF3*, *TREML1*, *P2RX1*, *CD70*, *CHRNA6*, *KLRC2*, and *CPS1* genes were identified (**Fig. 1E** and **1F**). Then, these genes were subjected to univariate Cox regression and stepwise regression to determine the final TME-related genes using minimum AIC as the criterion. The model consisting of *GCSAML*, *APOC4-APOC2*, *CHRDL1*, *GPR1*, *CPS1*, *IKZF3*, *P2RX1*, *CD70*, and* KLRC2* had a minimal AIC value (AIC = 1768.2, Table S1). Therefore, we developed the final risk model, called the tumor microenvironment-related index (TMEindex):

TEMindex = 0,93117 x GCSAML - 1.75862 x APOC4 - APOC2 + 0.116545 x CHRDL1 + 0.41756 x GPR1 + 0.06990 x CPS1 - 0.36391 x IKZF3 - 0.36001 x P2RX1 + 0.47444 x CD70 + 0.54178 x KLRC2

### The TMEindex exhibits clinical and prognostic implications

**Figure 2:**
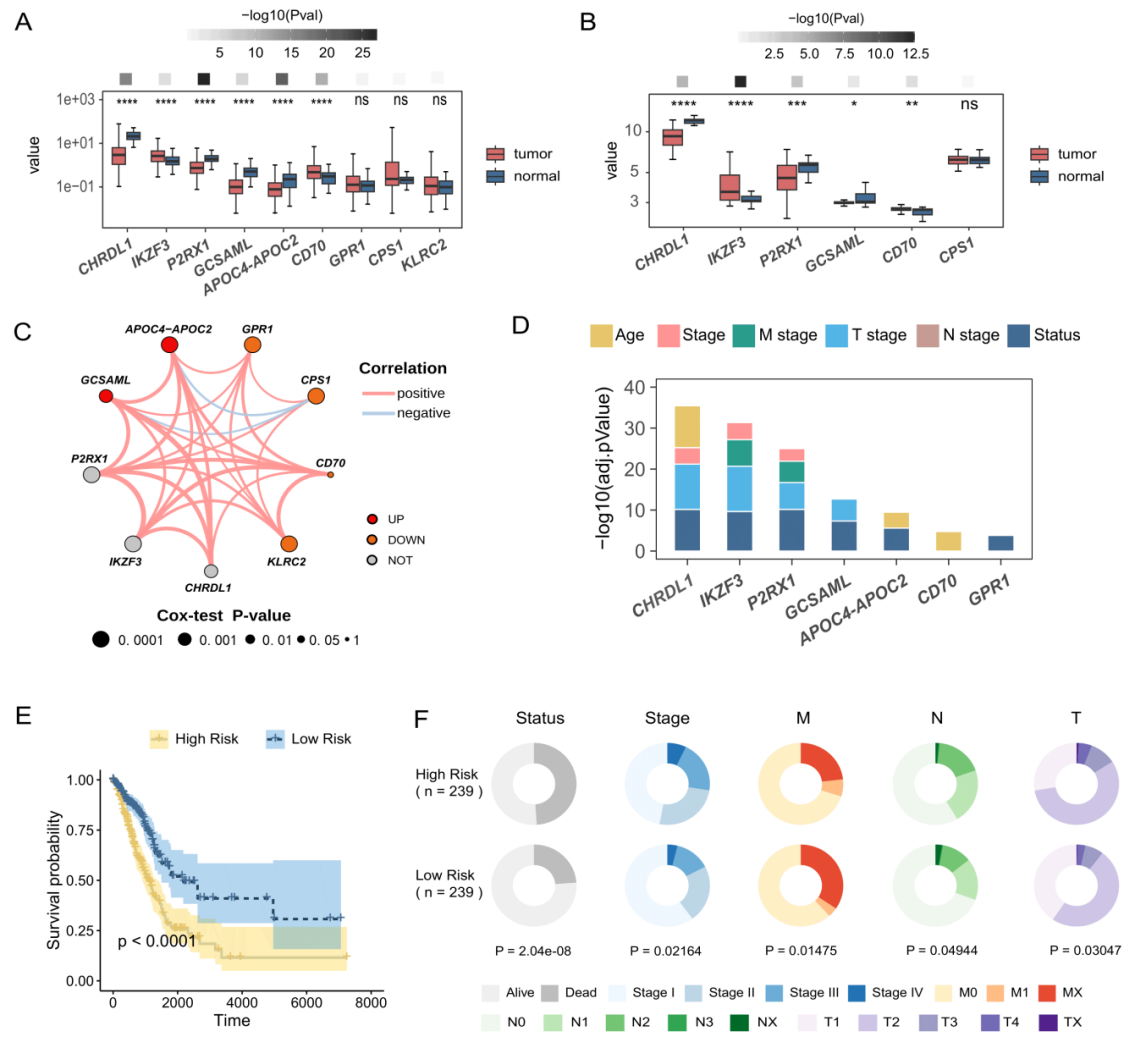
FIGURE 2: TME-related genes and the TMEindex exhibit prognostic and clinical implications. **(A)** Boxplots showing the differences in the expression of TME-related genes between tumor and tumor adjacent tissues in TCGA-LUAD. **(B)** Boxplots showing the differences in the expression of TME-related genes between tumor and tumor adjacent tissues in GSE101929. **(C)** Correlation network of the TME-related genes. The size of node indicates the statistical significance by univariate Cox regression analysis. **(D)** Barplot showing the association of the TME-related genes with clinical information. Kruskal-Wallis test was used to conduct the statistical test. **(E)** Survival curves of the high- and low-TMEindex group. Log-rank test was used to conducted the statistical test. **(F)** Differences in the clinical information between the high- and low-TMEindex group. Chi-square test was used to perform the statistical test. *P < 0.05, **P < 0.01, ***P < 0.001 and ****P < 0.0001. ns, not significant.

Next, we correlated the genes included in the TMEindex with tumorigenesis of LUAD. In TCGA-LUAD cohort, the expressions of *CHRDL1*, *P2RX1*, *GCSAML* and *APOC4-APOC2* in tumor tissues were significantly lower than those in normal tissues, indicating the tumor-suppressing role of these genes in LUAD. Meanwhile, the expressions of *IKZF3* and *CD70* were significantly higher in tumor tissues than those in normal tissues (**Fig. 2A**). Additionally, similar results were detected in GSE101929 dataset (**Fig. 2B**). Interestingly, we found that most of these gene signatures were positively related to each other (**Fig. 2C**), indicating their joint role in regulating tumor pathology. Together, these results highlight the potential association of genes included in our risk model with LUAD tumorigenesis.

Next, we sought to explore the association of the TMEindex with clinical phenotypes and patient outcome in LUAD. First, we found that the expression of TME-related genes was significantly correlated with multiple clinical parameters, such as age, tumor stage, and tumor size (**Fig. 2D**, adjusted *P* < 0.05). Furthermore, according to the median score, patients were divided into two groups, the high-TMEindex group and the low-TMEindex group. Survival analysis showed that patients in the high-TMEindex group exhibited a significantly shorter overall survival (OS) (**Fig. 2E**, *P* < 0.0001). Moreover, we compared multiple clinical parameters between the two groups. The results showed a significantly higher proportion of patients with advanced LUAD in the high-TMEindex group than in the low-TMEindex group (**Fig. 2F**, *P* < 0.05). Therefore, the TMEindex has profound clinical and prognostic implications.

### Patients in the high-TMEindex group present immunosuppressive TME

**Figure 3:**
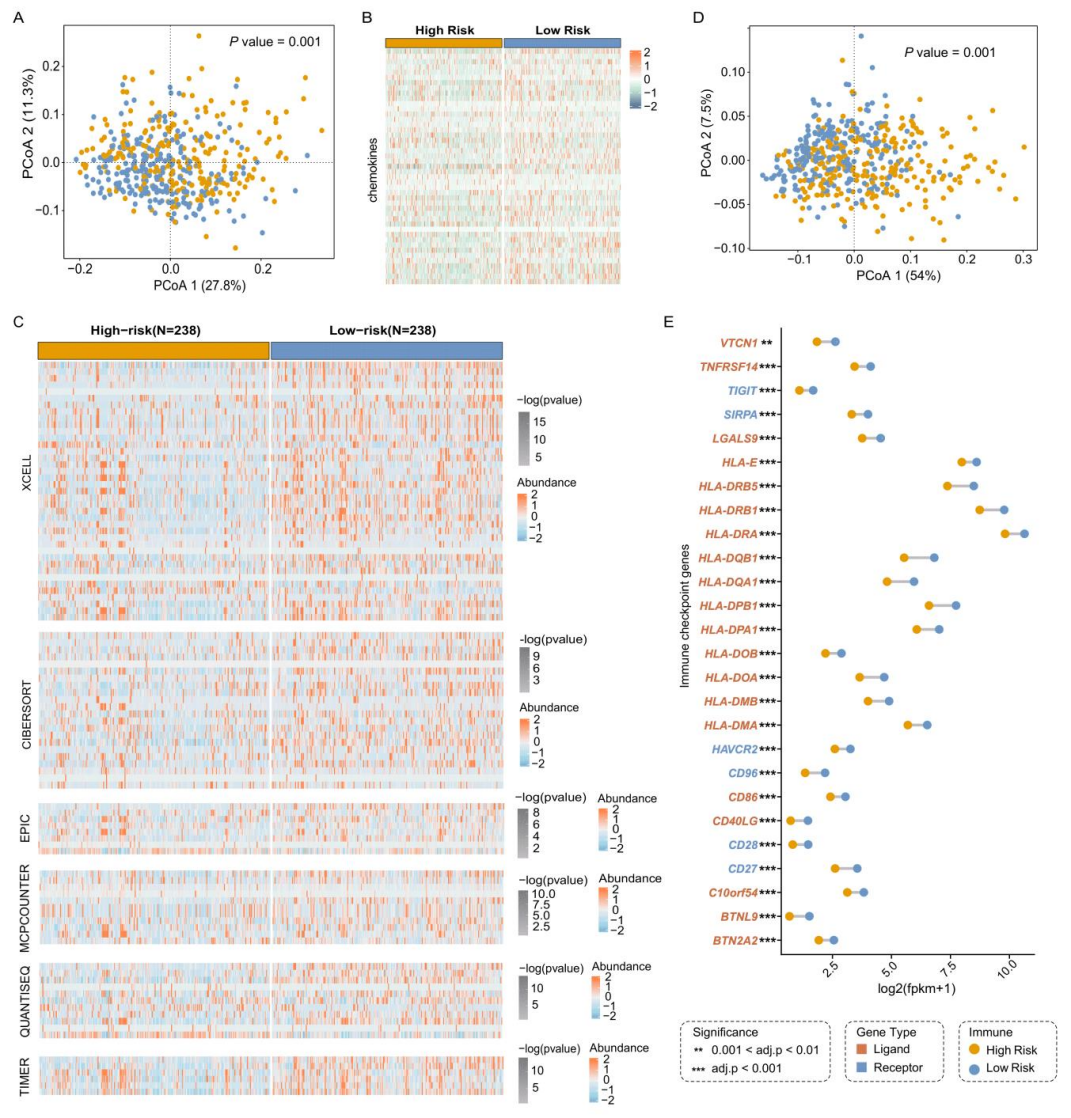
FIGURE 3: High-TMEindex group showed immunosuppressive characteristics compared with low-TMEindex. **(A)** PCoA showing the differences in the expression of chemokines between the high- and low-TMEindex. **(B)** Heamap showing the expression of chemokines in the high- and low-TMEindex. **(C)** Heatmap showing the normalized abundance of various immune cells in the high- and low-TMEindex. **(D)** PCoA showing the differences in the expression of immune checkpoint genes between the high- and low-TMEindex. **(E)** Barbell plot showing the difference in the expression of immune checkpoint genes between the two groups. Statistical tests were performed using Wilcoxon test. **P < 0.01 and ***P < 0.001.

Immune cells can be directly or indirectly involved in the immune response, affecting tumor growth and therapeutic response 31. Chemokines have the ability to induce directed chemotaxis of nearby reactive cells, which can recruit immune cells into tumors 32. We detected that the expression of chemokines in the high-TMEindex group was significantly lower than that in the low-TMEindex group (**Fig. 3A** and **3B**). Next, we analyzed the profile of infiltrating immune cells inferred by multiple approaches, including xCELL, CIBERSORT, EPIC, MCPcounter, QuanTIseq and TIMER. Intriguingly, compared with the low-TMEindex group, the high-TMEindex group had a significantly lower degree of immune cell infiltration (**Fig. 3C**). In addition, we found a clear divergence of the expression of immune checkpoint-related genes (ICGs) (**Fig. 3D**, *P* = 0.001). Specifically, we identified 26 ICGs with significantly higher expression in the low-TMEindex group, such as *CD86 *and *HLA-E* (**Fig. 3E**, adjusted *P* < 0.05). In conclusion, our prognostic model accurately delineates the degree of immune infiltration in patients with LUAD, and the difference in immune infiltration between the two groups explains the significant difference in prognosis.

### Intratumoral microbiome is closely related to the TMEindex

**Figure 4:**
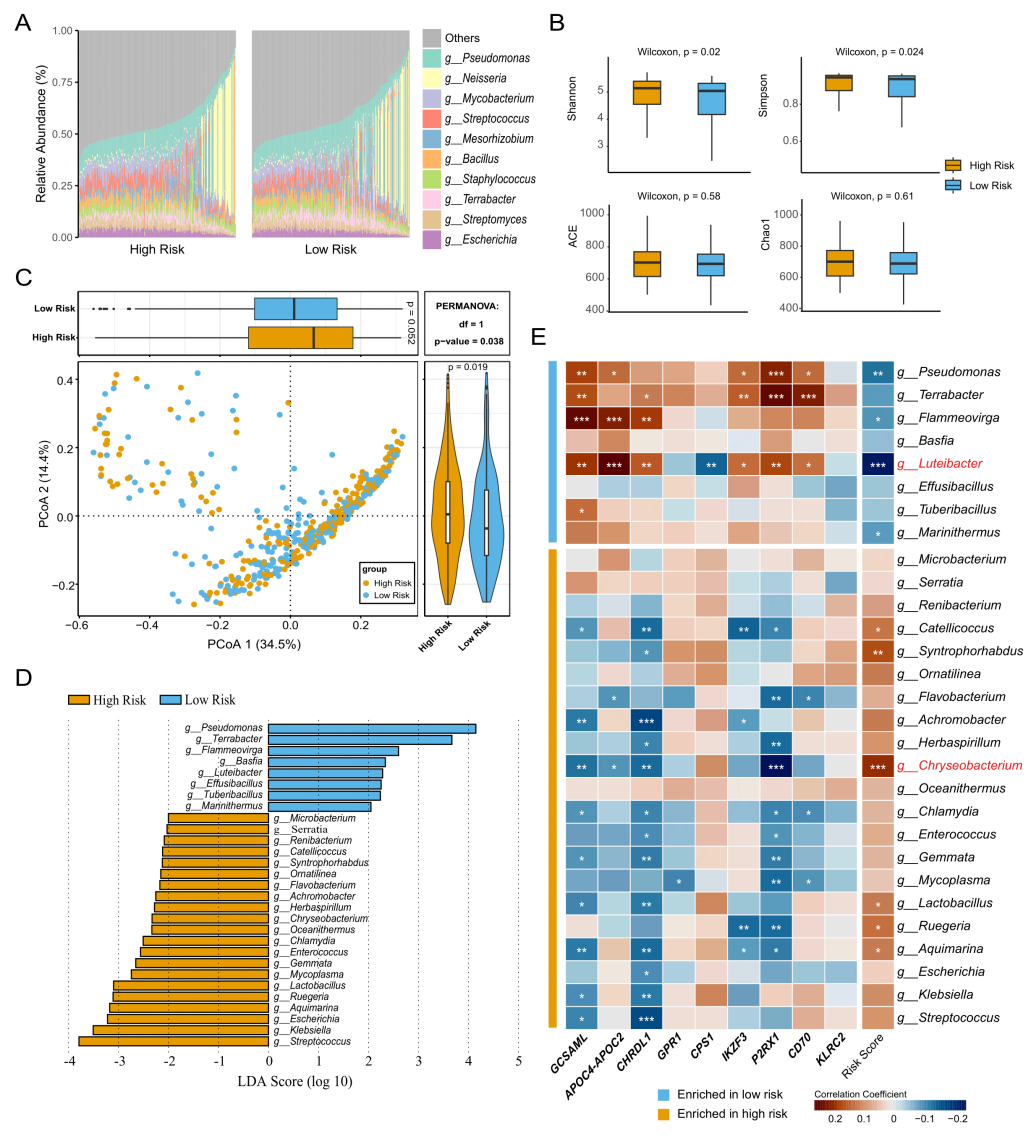
FIGURE 4: Association of the TMEindex with intratumoral microbiome. **(A)** Barplot showing the relative abundance of top ten genera in the high- and low-TMEindex groups. **(B)** Boxplot showing the differences in alpha diversity of microbial community between the high- and low-TMEindex groups. **(C)** PCoA showing the difference in microbiota composition between the high- and low-TMEindex groups. **(D)** Microbial biomarkers at the genus level identified by LEfSe. **(E) **Heatmap showing the Spearman correlation between the microbial biomarkers and TME-related genes and the TMEindex. *P < 0.05, **P < 0.01 and ***P < 0.001.

The microbiota can regulate anti-tumor immunity through its interaction with the TME and influence the efficacy of cancer immunotherapy 33. We comprehensively characterized the intratumoral microbial community profile in patients with LUAD. At the genus level, in both two groups, genus *Pseudomonas* had the highest relative abundance, followed by *Neisseria *and *Mycobacterium *(**Fig. 4A**). The Shannon and Simpson indices of the high-TMEindex group were significantly higher than those of the low-TMEindex group, and there was no significant difference in the ACE and Chao1 indices between the two groups (**Fig. 4B**, *P* < 0.05). In addition, we also observed a divergence of the microbial community at the genus level between the two groups (**Fig. 4C**, *P* = 0.038). Next, Linear discriminant analysis (LDA) effect size (LEfSe) analysis was performed to identify the microbial biomarkers. A total of 29 genera were identified, of which eight were enriched in the low-TMEindex group and 21 were more abundant in the high-TMEindex group (**Fig. 4D**).

Next, we explored the relationship between the 29 microbial biomarkers with our prognostic model. Interestingly, we observed that the microbes enriched in the low-TMEindex group were negatively correlated with the TMEindex score and positively correlated with the TME-related genes. On the contrary, the microbes enriched in the high-TMEindex group were positively correlated with the TMEindex score and negatively correlated with the TME-related genes (**Fig. 4E**). Specifically, the genus *Luteibacter*, enriched in the low-TMEindex group, was significantly negatively correlated with the TMEindex score. In addition, the genus *Chryseobacterium*, enriched in the high-TMEindex group, was significantly positively correlated with the TMEindex scores. In summary, our results suggest that intratumoral microbes closely interact with the TME and have the potential to be integrated into the prognostic model.

### The TMEindex can inform immunotherapy and drug sensitivity

**Figure 5:**
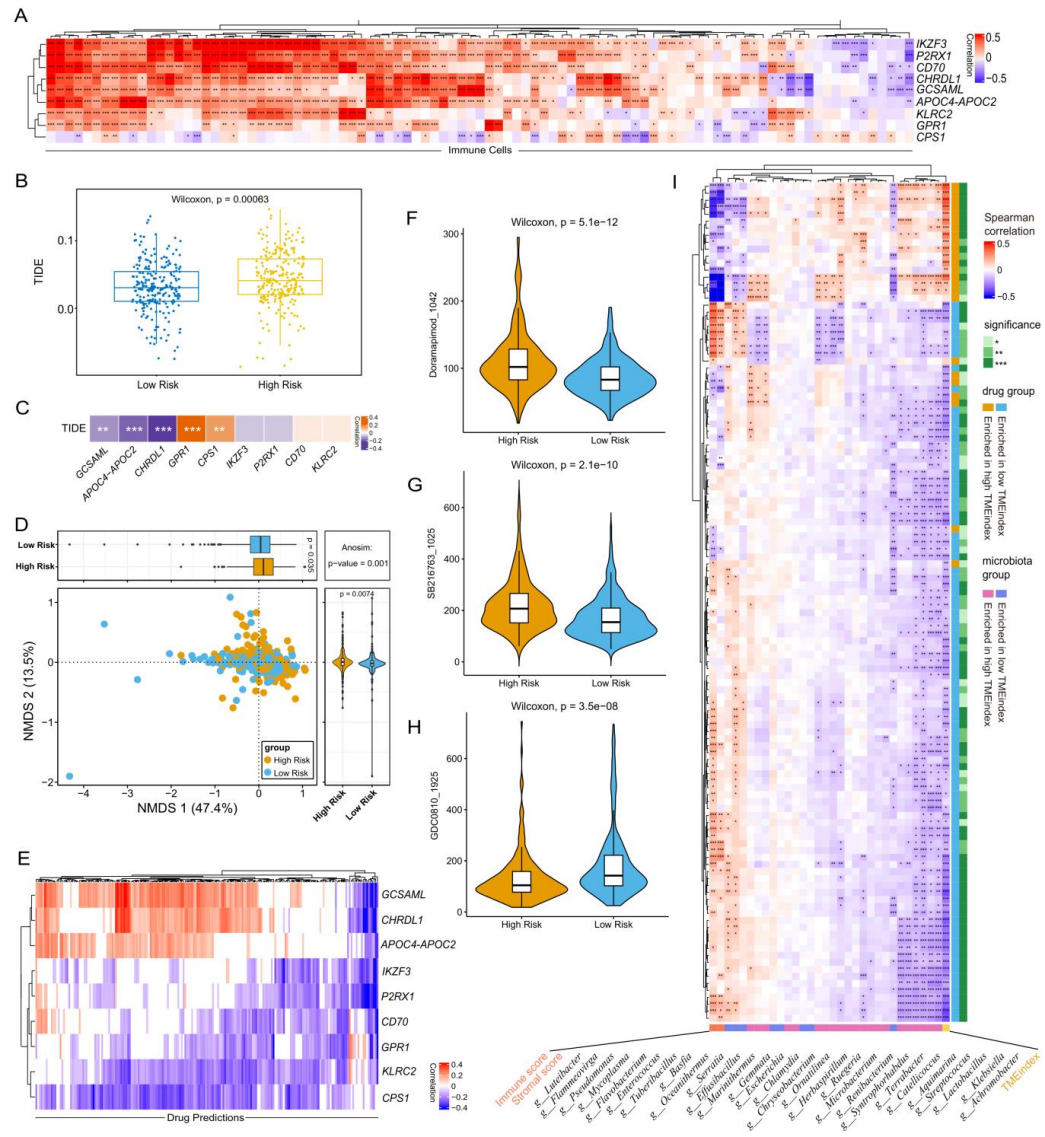
FIGURE 5: Potential role of the TMEindex in immunotherapy and conventional therapy. **(A)** Heatmap showing the association of the expression of TME-related genes with immune cells. **(B)** Boxplot showing the difference in the TIDE value between the high- and low-TMEindex groups. **(C)** Heatmap showing the association of the expression of the TME-related genes with TIDE value. **(D)** PCoA showing the differences in IC50 values of drugs between the two groups. **(E)** Heatmap showing the association of the expression of the TME-related genes with drug sensitivity. **(F-H)** Boxplots showing the differences in sensitivity of three drug candidates between the two groups. **(I)** Heatmap showing the association of the drug sensitivity with the TME characteristics, microbial biomarkers, and the TMEindex.

The application of immunotherapy to cancer has high clinical value, but only a subset of patients show response to immunotherapy 34. The degree of immune invasion can usually reflect the prognosis and immune response of the tumor 35. First, we found that the abundance of immune cell populations was significantly correlated with the expression of the TME-related genes (**Fig. 5A**). Next, we observed that the Tumor immun dysfunction and exclusion (TIDE) score of the high-TMEindex group was significantly higher than that in the low-TMEindex group (**Fig. 5B**, *P * < 0.05, Table S2), potentially indicating that patients in the high-TMEindex group showed adverse response to immunotherapy. Moreover, we detected that the expression of *GCSAML*, *APOC4−APOC2*, *CHRDL1* was significantly positively correlated with the TIDE score, while the expression of *GPR1* and *CPS1* was significantly negatively correlated with the TIDE score (**Fig. 5C**).

Next, we sought to explore the difference in biological function between the two groups. The DEGs between the high- and low-TMEindex were mainly enriched in hormone activity regulation, hormone metabolism, extracellular matrix organization, antibacterial humoral immune response, potential involvement in the interaction between neuroactive substances and their receptors, as well as steroid hormone biosynthesis (Fig. S1A-S1C).

Considering the importance of combination therapy in the treatment of LUAD, drug sensitivity analysis was conducted. The IC50 value of 198 drugs for each patient was predicted using R package "oncoPredict". We observed a clear divergence in drug sensitivity between the high- and low-TMEindex group (**Fig. 5D**, *P* = 0.001). We further explored the relationship between drug sensitivity and expression of the TME-related genes, and found that *IKZF3*, *P2RX1*, *CD70*, *GPR1*, *KLRC2*, and *CPS1* were potently associated with most of drugs (**Fig. 5E**). Specifically, we observed that patients in the high-TMEindex group could benefit from GDC0810, while Doramapimod and SB216763 were fit for patients in the low-TMEindex group (**Fig. 5F-5G**). Additionally, we comprehensively investigated the association of the TME characteristics, microbial biomarkers, and TMEindex with drug sensitivity, respectively (**Fig. 5I**). By Spearman correlation analysis, we found that drugs enriched in the high-TMEindex group was mainly positively correlated with the TMEindex and the microbial markers enriched in the high-TMEindex group (**Fig. 5I**). In addition, stromal score, immune score and microbial biomarkers enriched in the low-TMEindex showed the opposite relationship with drug sensitivity. Consequently, these results demonstrate that the TMEindex can identify patients fit for immunotherapy and can predict patients' sensitivity to conventional drugs.

### Integrating microbial biomarkers and the TME characteristics for prognosis prediction

**Figure 6:**
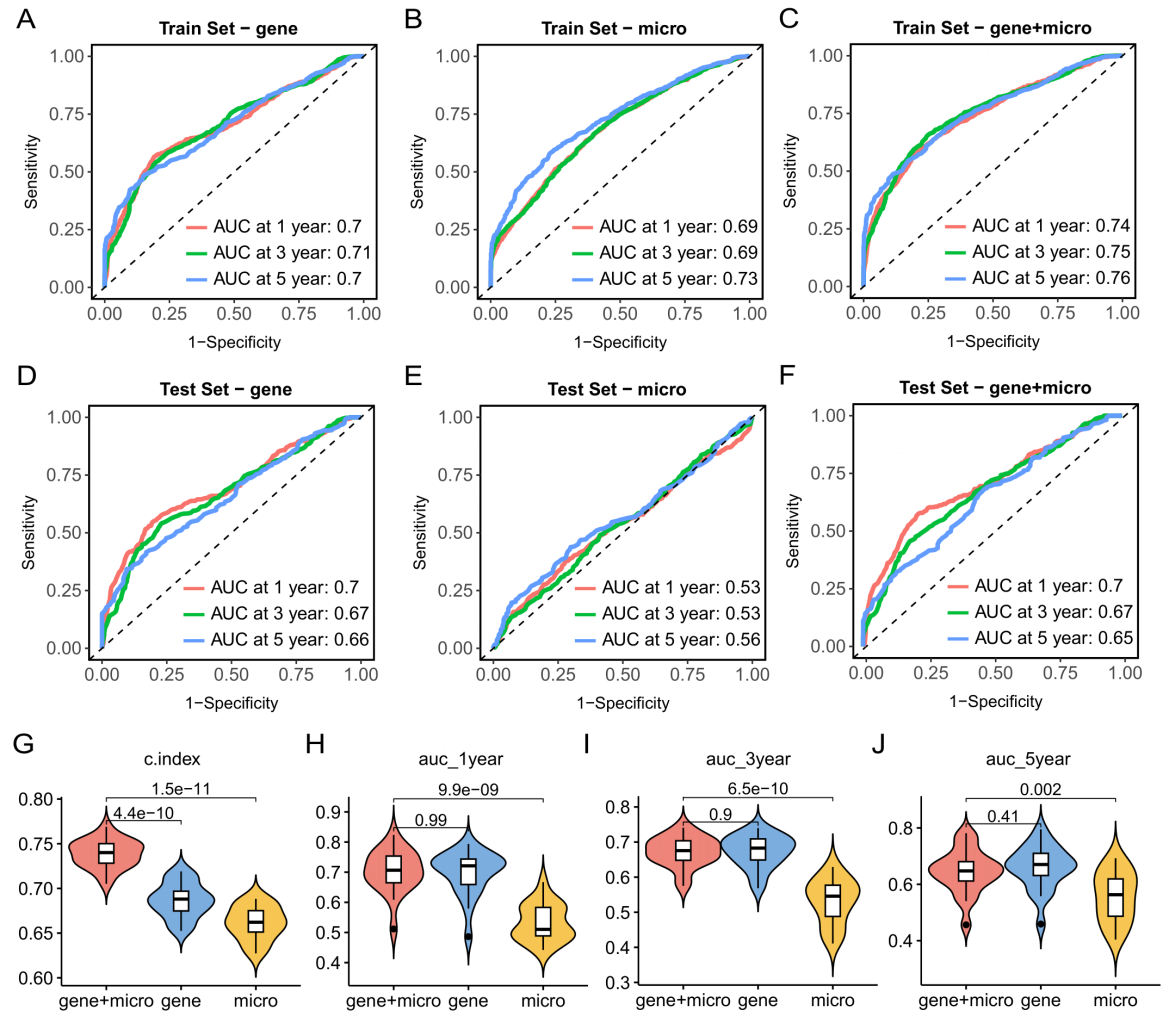
FIGURE 6: Assessment of the survival prediction ability of the TME-related genes and intratumoral microbiome. **(A-C) **Average ROC curves for predicting 1-, 3-, and 5-year survival on training set, based on TME-related genes, microbial biomarkers, and their combination, respectively. **(D-E)** Average ROC curves for predicting 1-, 3-, and 5-year survival on testing set. (G-J) Boxplots showing the differences in mean C index and the mean AUC among the three models. Statistical tests were performed using Wilcoxon test.

Although the interaction between intratumoral microbiota and the TME has been revealed, the fusion of the two to establish a prognostic model has not yet been implemented. Samples of TCGA-LUAD were assigned to the training set at a ratio of 70% and to the testing set at a ratio of 30%, and the partitioning and training process was repeated 20 times. First, Cox risk proportional regression model was constructed based on the TME-related genes, microbial biomarkers, and combination of the two on the training set, respectively (**Fig. 6A-6C**). The mean The Area Under the ROC curve (AUC) of the model for predicting 1-, 3-, and 5-year survival on the testing set was 0.7, 0.67, and 0.66, respectively (**Fig. 6D**). Secondly, Cox risk proportional regression model was established based on microbial biomarkers, and the average AUC of 1-, 3-, and 5-year survival rates predicted on the testing set were 0.53, 0.53, and 0.56, respectively (**Fig. 6E**). Finally, the combination of the TME-related genes and microbial biomarkers were input into the Cox model, and the model predicted the mean AUC of 1-, 3-, and 5-year survival on the testing set to be 0.7, 0.67, and 0.65, respectively (**Fig. 6F**). We aggregated the C index and AUC values of all models and found that the model established by mixed features had a better effect (**Fig. 6G-6J**, Table S3-S5). At this point, we finally determined the final model, called intratumoral microbiome-modified TMEindex (IMTMEindex) (Table S6)：

IMTMEindex = 0.43657 x GCSAML - 0.24064 x APOC4 - APOC2 + 0.16043 x CHRDL1 + 0.15889 x GPR1 + 0.14000 x CPS1 - 0.30345 x IKZF3 + 0.26408 x CD70 + 0.29906 x KLRC2 - 0.20135 x g_Terrabacter + 0.14646 x g_Effusibacillus + 0.19592 x Marinithermus + 0.27673 x g_Renibacterium - 0.62063 x g_Achromobacter + 0.24935 x g_Herbaspirillum + 0.19307 x g_Chlamydia - 4.33628 x g_Lactobacillus - 0.20560 x g_Aquimarina + 0.61934 x g_Klebsiella + 0.22734 x g_Streptococcus

## DISCUSSION

The intratumoral microbiome is related to the occurrence, development and metastasis of tumors, and may even affect the prognosis of patients 36. Here, we constructed a 9-gene signature to characterize the TME. Besides, we built a scoring system TMEindex based on these nine genes. The results showed that clinical information, response to immunotherapy, drug sensitivity, and intratumoral microbiota of patients with LUAD were significantly different between the high- and low-TMEindex groups. We identified specific microbial biomarkers that could affect the prognosis of patients with LUAD, and based on this, we established the IMTMEindex. It was found that combining the characteristics of intratumoral microbiota and the TME signatures was more effective in predicting the prognosis of patients.

LUAD-related genes are being studied. Deng *et al*. reported that *CHRDL1* expression showed a good ability to distinguish LUAD tissue from normal samples. Low *CHRDL1* is associated with adverse clinicopathological features and poor OS 37. Yang *et al*. found that low expression of *P2RX1 *associated with poor OS and decreased immune infiltration 38. Wen *et al*. reported that P2RX1 is a risk factor that has an inverse relationship with risk score 39. In the process of extracting TME characteristics, nine TME-related genes were screened by LASSO-Cox regression. Among them, the expression of *CHRDL1*, *P2RX1*, *GCSAML*, and *APOC4-APOC2* in the tumor region was significantly lower than that in the normal region. This suggested that these genes may be protective genes. These nine genes were also associated with clinical characteristics such as age and TNM stage. These results are consistent with previous studies.

Some studies have shown that the infiltration level of immune cells in TME is directly related to immune efficacy 40,41,42,43. Our results showed that the infiltration degree of T lymphocytes (including CD4+ and CD8+T cells), B lymphocytes, NK cells and dendritic cells in the high-TMEindex group was significantly lower than that in the low-TMEindex group. ICGs play pivotal roles in tumor immune microenvironment (TIME) 44. In the comparison of immune checkpoint gene expression between the two groups, most ICGs, especially HLA families, also showed lower expression in the high-TMEindex group. We found that patients in the high-TMEindex group had a suppressed immune system compared to those in the low-TMEindex group. Lower TIDE score indicated that patients in the low-TMEindex group might respond better to immunotherapy.

We found that there may be some interaction between microorganisms and the TME. Surprisingly, the microbial biomarkers enriched in the low-TMEindex group were negatively correlated with the TMEindex score, while the microbial biomarkers significantly enriched in the high-TMEindex group were positively correlated with the TMEindex score. In particular, relevant conclusions have been found not only in our study, but also in previous studies on *Luteibacter* and *Chryseobacterium*. Deng *et al*. found that in patients with LUAD, the abundance of *Luteibacter* was lower in the stage III-IV group 45. Zheng *et al*. found that among tumor-resident microorganisms in patients with non-small cell lung cancer (NSCLC), the abundance of *Chryseobacterium* is different in tumor and adjacent tissue. *Chryseobacterium* was significantly increased in a group of tumor-bearing mice that inhaled bronchoalveolar fluid (BAL) collected from patients with NSCLC 46. Ye *et al*. found that Pachymaran (PCP), a drug ingredient, can intervene to significantly reduce the abundance of *Chrysobacterium*, thereby reducing immunosuppressive lung injury 47. Based on these studies, our study uncovered the prognostic implications of tumor-resident microbes in patients with LUAD and demonstrated the close crosstalk between the TME and tumor-resident microbes.

At present, in order to accurately predict the prognosis of patients with LUAD, many risk models have been established. Yang *et al*. used the TCGA database to screen long non-coding RNA-associated immune genes (lrRIGs) associated with LUAD prognosis, and then constructed a tag consisting of nine prognostic genes. This risk score showed excellent performance in predicting the OS rate 48. Wu *et al*. constructed a TMEindex model to predict OS in patients with LUAD. Functionally, TMEindex score is related to immune response in patients with LUAD 49. Deng *et al*. established a prognosis score derived from the abundance of microorganisms in tumors of patients with LUAD, namely lung resident microbial score (LMS), and evaluated its relationship with LUAD prognosis and immune invasion 45. In our study, a risk model scoring system IMTMEindex, was established with mixed TME-related genes and microbial biomarkers. Moreover, the C index of IMTMEindex was significantly higher than models based solely on the TME characteristics. IMTMEindex may be used as a prognostic predictor of LUAD and provide valuable information for precise treatment.

Current studies are mostly based on bulk sequencing data. In the future, single-cell sequencing, spatial transcriptomics and other technologies can be combined to analyze the dynamic interaction network of microorganisms with immune cells and stromal cells within tumors, reveal how microbes directly affect TME function through metabolites (such as short-chain fatty acid, tryptophan derivatives) or immunomodulatory molecules (such as PD-L1). For example, specific bacterial species may contribute to immunosuppression by inducing polarization of M2 macrophages, a process that can be precisely targeted by single-cell techniques. In addition, microbial characteristics may become a new dimension of stratified treatment. For example, in patients with microbiota-driven immunosuppressive TME, combinations of antibiotics (such as narrow-spectrum antibiotics against specific strains), probiotic supplementation, or phage therapy, may be indicated, synergism with ICIs. Prospective clinical trials are needed to verify the effect of microbial regulation on the improvement of ICIs response rate.

There are several limitations in our study. First, the intratumoral microbiome data in our study have limited resolution and only genus-level annotations. In the future, metagenomic sequencing technology will be applied to lung tumor samples to obtain intratumoral microbial information at the species and even strain level. Second, independent data on the intratumoral microbiome abundance are not available. However, it is important to note that host transcriptome samples and matched intratumoral microbiome samples from 478 patients were included in our study, already a relatively large sample size to support our findings. Future accumulations of larger lung tumor microbiome data will refine our results. Finally, our results are largely based on correlations and hypotheses, and more *in vitro* and *in vivo* trials are needed in the future to explore the mechanisms of interaction between the tumor microbiota and the TME, as well as causality.

Overall, our study investigated the interaction between the TME signatures and intratumoral microbiota in LUAD. We found that tumor-resident *Luteibacter *and *Chryseobacterium *were substantially associated with survival risk of patients with LUAD. Besides, the combination of TME characteristics and intratumoral microbiome introduced additionally prognostic implications. IMTMEindex was a promising biomarker for predciting prognosis and therapeutic opportunities in LUAD. Further studies are needed to clarify the regulatory mechanisms of specific intratumoral microbes on LUAD microecology.

## MATERIALS AND METHODS

### Data acquisition

A total of 478 patients with LUAD were included in our study. Each patient had a host transcriptome sample and a matched intratumoral microbiome sample. The gene expression data was obtained from TCGA (https://portal.gdc.cancer.gov/). Clinical metadata of patients was downloaded from UCSC Xena (http://xena.ucsc.edu . The majority of the samples were primary solid tumors, with a small number of recurrent or metastatic tumors. The sample covers stage I-IV patients, with the highest proportion of stage I (about 40%) and the lowest proportion of stage IV (about 10%). Intratumoral microbiome abundance data was obtained from the work by Narunsky-Haziza *et al*. and was downloaded from https://github.com/knightlab-analyses/mycobiome. The quality control and taxonomic annotation were detailed in the original paper 50. Independent validation dataset GSE101929 was downloaded from The Gene Expression Omnibus (GEO) dataset (https://www.ncbi.nlm.nih.gov/geo/).

### Construction of prognostic model

The DEGs were identified with R package "DESeq2" 51. Then, Weighted Gene Co-expression Network Analysis (WGCNA) was performed using R package "WGCNA" to identify genes associated with the stromal and immune scores 52. Subsequently, least absolute shrinkage and selection operator (LASSO) 53 regression algorithm was conducted to further screen. Then, the multivariate Cox proportional hazards regression analysis was performed to calculate the coefficient of each gene. Therefore, the final prognostic model, called the tumor microenvironment-related index (TMEindex), was as follows:

TMErisk = 0.93117 x GCSAML - 1.75862 x APOC4 - APOC2 + 0.11645 x CHRDL1 + 0.41756 x GPR1 + 0.06990 x CPS1 - 0.36391 x IKZF3 - 0.36001 x P2RX1 + 0.47444 x CD70 + 0.54178 x KLRC2

### Immune analyses

Tumor immun dysfunction and exclusion (TIDE) algorithm simulates the mechanism of tumor immune escape by inducing T cell dysfunction in tumors with high infiltrating cytotoxic T lymphocytes (CTL) and preventing T cell infiltration in tumors with low infiltrating cytotoxic T lymphocytes, thereby predicting patients' immunotherapy response 54. TIDE algorithm was used to evaluate the possibility of tumor immune escape in gene expression profiles of tumor samples. Patients with LUAD were divided into two groups according to the expression levels of CTL markers (*CD8A*, *CD8B*, *GZMA*, *GZMB*, and *PRF1*). In patients with high CTL marker levels, the correlation between the expression profile of each patient and the signature of T cell dysfunction was used as the TIDE score. In patients with low CTL marker levels, the correlation between the expression profile of each patient and T cell rejection signature was taken as the TIDE score.

Estimation of STromal and Immune cells in Malignant Tumor tissues using Expression data (ESTIMATE) algorithm 55 was used to obtain the immune score and stromal score of patients with LUAD. The CIBERSORT 56, TIMER 57, xCell 58, EPIC 59, quanTIseq 60 and MCPcounter 61 tools were used to assess the abundance of immune cells in the TME.

### Microbial analyses

The alpha diversity of microbial communities was measured by the Shannon, Simpson, ACE and Chao1 indices. The beta diversity analysis was carried out by Principal Co-ordinates Analysis (PCoA) based on Bray-Curtis distance matrix. Linear discriminant analysis (LDA) effect size (LEfSe) was used to identify the genera with significant differences in abundance 62.

### Other statistical analysis

All statistical analyses were performed using RStudio (version 4.3.2). K-M curves and log-rank tests were used to plot survival curves and compare survival differences. Wilcoxon test was used to explore the differences between the high- and low-TMEindex groups in feature genes, TIDE scores, immune checkpoint genes and other continuous variables. Chi-square test was used to compare the differences in clinical traits between the high- and low-TMEindex. Kruskal-Wallis test was used to explore the relationship between feature genes and clinical traits. The gene expression profile, microbial community, and drug sensitivity between the high- and low-TMEindex groups were delineated using Principal Coordinate Analysis (PCoA) and Permutational Multivariate Analysis of Variance (PERMANOVA). Spearman correlation analysis was used to calculate correlations between feature genes, between feature genes and immune cells, between feature genes and drug IC50 values, and between immune cells and microbial abundance. All comparisons reached statistical significance when the two-tailed adjusted p-value was less than 0.05.

### Availability of data and material

The data supporting the findings of this study are available in Materials and methods. Further inquiries can be directed to the corresponding author.

## AUTHOR CONTRIBUTION

F Z (Fei Zhao): Formal analysis, Writing—original draft, Writing—review & editing, Validation. L W (Lei Wang): Software, Methodology, Formal analysis. D D (Dongjie Du) and H Z (Heaven Zhao): Data curation, Writing—review & editing, Software. G T (Geng Tian): Software, Writing—review & editing. J L (Jingwu Li): Data curation, Writing—review & editing. Y L (Yufeng Li): Software, Writing—review & editing. Y L (Yankun Liu): Software, Writing—review & editing. Z W (Zhiwu Wang): Conceptualization, Writing-review & editing. D L (Dasheng Liu): Software, Writing—review & editing. L J (Lei Ji): Conceptualization, Writing-review & editing. H Z (Hong Zhao): Supervision, Project administration. All authors have read and approved the final manuscript.

## CONFLICT OF INTEREST

The authors declare no competing interests.

## SUPPLEMENTAL MATERIAL

Click here for supplemental data file.

All supplemental data for this article are available online at www.microbialcell.com/researcharticles/2025a-zhao-microbial-cell/.
