# Supplementary material for: Tumor microenvironment signatures enhances lung adenocarcinoma prognosis prediction: Implication of intratumoral microbiota
Source: Microb Cell. 2025 Aug 11;12:182–94. doi: 10.15698/mic2025.08.855 (PMC12373402; doi:10.15698/mic2025.08.855)
Supplement: Supplementary file 1 [file mic-12-182-s01.pdf]

Tumor microenvironment signatures enhances lung adenocarcinoma prognosis prediction: Implication of intratumoral microbiota

Fei Zhao[1#], Lei Wang[2,3,4#], Dongjie Du[5], Heaven Zhao[6,7], Geng Tian[6,7], Yufeng Li[2,3,8], Yankun Liu[2,8,9], Zhiwu Wang[2,3,10], Dasheng Liu[11], Jingwu Li[2,3,12*], Lei Ji[6,7*], Hong Zhao[1*]

[1]School of Mathematical Sciences, Ocean University of China, Qingdao, 266100, China
[2]Hebei Key Laboratory of Molecular Oncology, Hebei, 063000, China
[3]Tangshan Key Laboratory of Cancer Prevention and Therapy, Hebei, 063000, China
[4]Department of Pathology, Tangshan People's Hospital, Hebei, 063000, China
[5]Department of Vascular Surgery, Hebei General Hospital, Shijiazhuang, 050051, China
[6]Geneis Beijing Co., Ltd., Beijing, 100102, China
[7]Qingdao Geneis Institute of Big Data Mining and Precision Medicine, Qingdao 266000, China
[8]The Cancer Institute, Tangshan People's Hospital, Hebei, 063000, China
[9]Tangshan Key Laboratory of Precision Medicine Testing, Hebei, 063000, China
[10]Department of Chemoradiotherapy, Tangshan People's Hospital, Hebei, 063000, China
[11]Department of Thoracic Surgery, Jiamusi Central Hospital, Jiamusi, 154000, China
[12]Department of Gastrointestinal Surgery, Tangshan People's Hospital, Hebei, 063000, China

[#]The authors contributed equally to this work
[*]Correspondence: Jingwu Li, E-mail: tslijingwu@163.com
Lei Ji, E-mail: jil@geneis.cn
Hong Zhao, E-mail : zhaohong@ouc.edu.cn

Supplementary figures

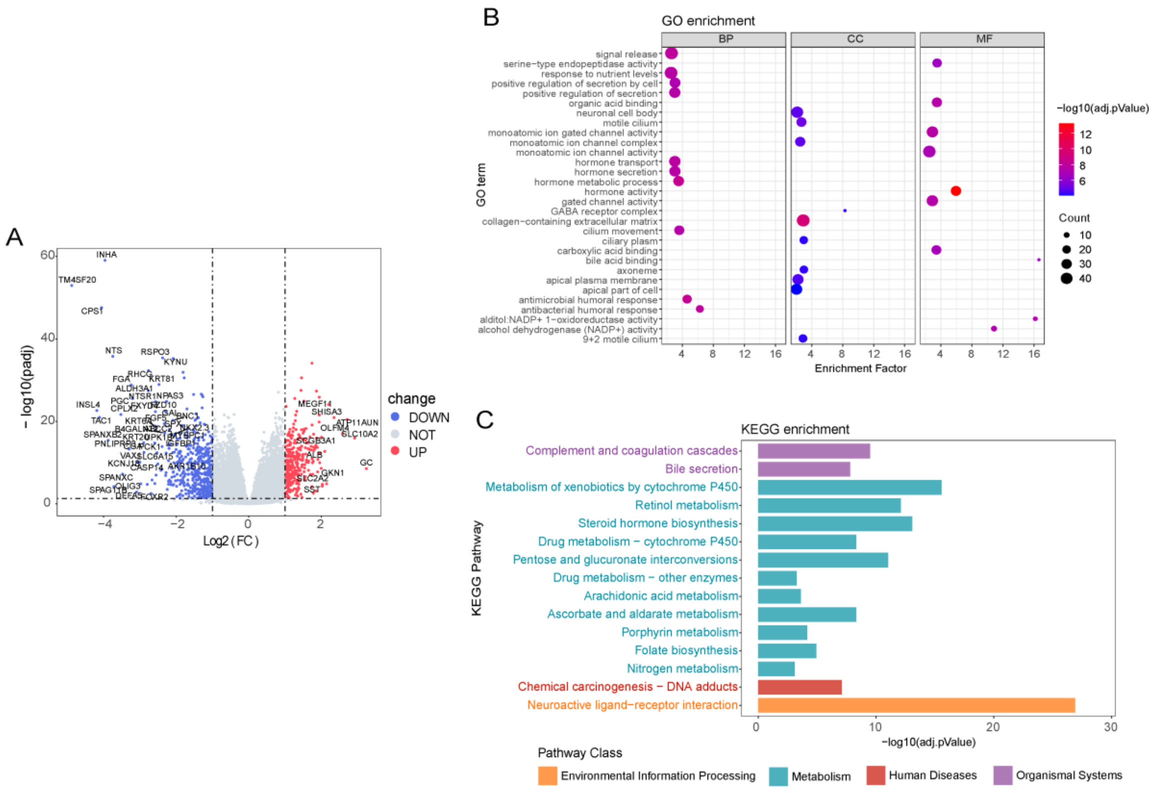

Supplementary Figure 1. Results of differential expression gene analysis and enrichment analysis of TMErisk.

(A) Volcanic maps showed differential genes in the high- and low-TMErisk groups. (B-C) GO and KEGG enrichment analysis of TMErisk.

Supplementary Table 1. The association of TME-related genes and LUAD mortality

	coef[a]	HR	lower CI of HR	upper CI of HR
GCSAML	-0.9311743	0.394090658	0.179735357	0.864089567
APOC4-APOC2	-1.758616259	0.172283094	0.045854782	0.647292679
CHRDL1	0.116446797	1.123497735	0.963813899	1.309637849
GPR1	0.417559656	1.518251974	1.134215008	2.03232107
CPS1	0.069895617	1.072396235	1.0106564	1.137907686
IKZF3	-0.363907437	0.694955519	0.536447382	0.900299246
P2RX1	-0.360012971	0.697667276	0.47354158	1.027870938
CD70	0.474444043	1.607120459	1.276493385	2.023383903
KLRC2	0.541781487	1.719066631	1.231165531	2.400319053

[a] The multivariable Cox regression model coefficient.
Abbreviations: HR, hazard ratiol; CI, confidence interva.

Supplementary Table 2. TIDE score of LUAD samples

	Dysfunction	Exclusion	CTL_group[a]	TIDE[b]	Scale_TIDE
TCGA-05-4249	0.041792176	0.04171769	Low_CTL	0.04171769	0.102364063
TCGA-05-4250	0.082456444	0.047392797	High_CTL	0.082456444	1.128754045
TCGA-05-4382	0.025515649	0.039621548	High_CTL	0.025515649	-0.305837244
TCGA-05-4384	0.077104832	0.02022673	Low_CTL	0.02022673	-0.439088558
TCGA-05-4389	-0.057459776	0.018200755	High_CTL	-0.057459776	-2.396356365
TCGA-05-4390	0.050556383	0.031892555	Low_CTL	0.031892555	-0.145174664
TCGA-05-4396	0.060910439	0.017702061	Low_CTL	0.017702061	-0.502696192
TCGA-05-4397	-0.113132386	0.049340792	Low_CTL	0.049340792	0.294423842
TCGA-05-4398	0.058467768	0.032912971	High_CTL	0.058467768	0.524372868
TCGA-05-4402	0.051562988	0.044371185	Low_CTL	0.044371185	0.169217386
TCGA-05-4403	-4.81E-05	0.014707086	Low_CTL	0.014707086	-0.578152888
TCGA-05-4405	0.071892844	0.087923163	Low_CTL	0.087923163	1.266484946
TCGA-05-4415	0.078785552	0.050901853	High_CTL	0.078785552	1.036267997
TCGA-05-4417	0.022322875	0.043098033	High_CTL	0.022322875	-0.386277374
TCGA-05-4418	0.076568513	0.018892784	High_CTL	0.076568513	0.980410935
TCGA-05-4420	-0.00874634	0.04401885	Low_CTL	0.04401885	0.160340496
TCGA-05-4422	0.018222173	0.020291714	High_CTL	0.018222173	-0.489592263
TCGA-05-4424	-0.006085855	0.031594806	Low_CTL	0.031594806	-0.152676289
TCGA-05-4425	0.01911295	0.04065052	High_CTL	0.01911295	-0.467149622
TCGA-05-4426	0.056536838	0.028546761	Low_CTL	0.028546761	-0.229470074
TCGA-05-4427	0.020902246	0.047743969	High_CTL	0.020902246	-0.422069319
TCGA-05-4430	0.022813761	0.059064366	High_CTL	0.022813761	-0.373909791
TCGA-05-4432	0.018820624	0.040790042	High_CTL	0.018820624	-0.474514618
TCGA-05-4433	-0.030756139	-0.013218415	High_CTL	-0.030756139	-1.72357326
TCGA-05-4434	0.045603444	0.036372448	High_CTL	0.045603444	0.200263454
TCGA-05-5420	-0.047858635	0.009015908	High_CTL	-0.047858635	-2.154461015
TCGA-05-5423	-0.074025547	0.015391832	High_CTL	-0.074025547	-2.813721642
TCGA-05-5425	-0.007115356	0.029797127	High_CTL	-0.007115356	-1.127957041
TCGA-05-5428	-0.067299578	0.032965826	Low_CTL	0.032965826	-0.118134219
TCGA-05-5429	-0.020781376	0.030125124	Low_CTL	0.030125124	-0.189704109
TCGA-05-5715	0.005266143	0.020962523	Low_CTL	0.020962523	-0.42055068
TCGA-35-3615	0.023351699	0.028292253	Low_CTL	0.028292253	-0.235882249
TCGA-35-4122	-0.054705748	0.022641709	High_CTL	-0.054705748	-2.326970182
TCGA-35-4123	-0.01068402	0.029915287	High_CTL	-0.01068402	-1.217867523
TCGA-35-5375	-0.072845195	0.011693705	High_CTL	-0.072845195	-2.783983339
TCGA-38-4626	0.043945105	0.01236568	Low_CTL	0.01236568	-0.637143295
TCGA-38-4627	0.001520963	0.10843742	Low_CTL	0.10843742	1.783330102
TCGA-38-4628	-0.032818166	0.036798947	Low_CTL	0.036798947	-0.021560887
TCGA-38-4630	0.007871415	0.071270304	Low_CTL	0.071270304	0.846925547
TCGA-38-4631	0.012904656	0.015922051	High_CTL	0.012904656	-0.623564108

TCGA-38-4632	-0.029383332	0.038077629	High_CTL	-0.029383332	-1.688986162
TCGA-38-6178	0.06761702	0.087060657	Low_CTL	0.087060657	1.244754611
TCGA-38-7271	0.011216765	0.032651426	High_CTL	0.011216765	-0.666089562
TCGA-38-A44F	0.069382705	0.029334378	High_CTL	0.069382705	0.799368546
TCGA-44-2655	0.017361477	0.033725656	Low_CTL	0.033725656	-0.098990736
TCGA-44-2657	-0.028634057	0.010914901	High_CTL	-0.028634057	-1.670108605
TCGA-44-2659	-0.008934983	0.050669519	High_CTL	-0.008934983	-1.173801512
TCGA-44-2661	-0.044340045	0.018211191	High_CTL	-0.044340045	-2.065812113
TCGA-44-3396	0.058888553	0.063048686	High_CTL	0.058888553	0.5349743
TCGA-44-3398	-0.0117327	0.043587103	High_CTL	-0.0117327	-1.24428843
TCGA-44-3917	-0.086547473	0.055930995	High_CTL	-0.086547473	-3.129204509
TCGA-44-3919	0.028712531	0.031890718	High_CTL	0.028712531	-0.225293587
TCGA-44-5643	0.051924362	0.0457425	High_CTL	0.051924362	0.359515438
TCGA-44-5644	0.110269224	0.067352338	Low_CTL	0.067352338	0.748214591
TCGA-44-6145	0.056720872	0.036181975	High_CTL	0.056720872	0.480360792
TCGA-44-6148	-0.023203937	0.027808705	Low_CTL	0.027808705	-0.248064985
TCGA-44-6774	0.053936123	0.164019756	Low_CTL	0.164019756	3.183695779
TCGA-44-6776	0.083595127	0.013141991	Low_CTL	0.013141991	-0.617584591
TCGA-44-6777	-0.058198092	0.027521345	Low_CTL	0.027521345	-0.255304839
TCGA-44-6778	-0.051669163	0.008998137	High_CTL	-0.051669163	-2.250465136
TCGA-44-6779	0.113498526	0.050747234	High_CTL	0.113498526	1.910841821
TCGA-44-7659	0.077790111	0.008459764	Low_CTL	0.008459764	-0.735550667
TCGA-44-7660	-0.035532524	0.026714674	Low_CTL	0.026714674	-0.275628471
TCGA-44-7661	0.082258853	0.066219473	High_CTL	0.082258853	1.123775857
TCGA-44-7662	0.049429162	0.107778395	High_CTL	0.049429162	0.296650264
TCGA-44-7667	0.043930257	0.055836854	High_CTL	0.043930257	0.158108443
TCGA-44-7669	0.026863016	0.036318156	High_CTL	0.026863016	-0.271891069
TCGA-44-7670	0.046024196	0.041700229	Low_CTL	0.041700229	0.10192415
TCGA-44-7671	0.079418369	0.02868113	Low_CTL	0.02868113	-0.22608471
TCGA-44-7672	0.054487744	0.0532415	High_CTL	0.054487744	0.424098386
TCGA-44-8117	0.072948333	0.07035212	Low_CTL	0.07035212	0.82379241
TCGA-44-8119	0.085700985	0.075686098	High_CTL	0.085700985	1.210498426
TCGA-44-8120	0.09245097	0.075056212	Low_CTL	0.075056212	0.942309374
TCGA-44-A479	0.009920051	0.064477273	High_CTL	0.009920051	-0.698759539
TCGA-44-A47A	0.019549892	0.029560964	High_CTL	0.019549892	-0.45614113
TCGA-44-A47B	0.107437295	0.036577721	High_CTL	0.107437295	1.758132525
TCGA-44-A47G	0.067714937	0.039726134	High_CTL	0.067714937	0.75735008
TCGA-44-A4SS	0.039647074	0.02275878	High_CTL	0.039647074	0.050196069
TCGA-44-A4SU	0.071765028	0.05802607	Low_CTL	0.05802607	0.513244519
TCGA-49-4486	0.070123816	0.010902552	Low_CTL	0.010902552	-0.674005986
TCGA-49-4487	0.054031983	0.056172967	High_CTL	0.054031983	0.412615754
TCGA-49-4488	0.000354495	0.050014691	High_CTL	0.000354495	-0.939758341
TCGA-49-4490	0.07475961	0.054896294	Low_CTL	0.054896294	0.43439158
TCGA-49-4494	0.089492341	0.024289197	High_CTL	0.089492341	1.306019519

TCGA-49-4501	-0.018674826	0.042322426	High_CTL	-0.018674826	-1.419191365
TCGA-49-4505	0.030593673	0.039428166	High_CTL	0.030593673	-0.17789928
TCGA-49-4506	0.076285251	0.01964254	High_CTL	0.076285251	0.973274306
TCGA-49-4507	0.118947607	0.024970156	High_CTL	0.118947607	2.048128347
TCGA-49-4510	0.074319311	0.022084185	Low_CTL	0.022084185	-0.392291036
TCGA-49-4512	-0.00511442	0.051481398	Low_CTL	0.051481398	0.348355203
TCGA-49-4514	0.032015624	0.030064856	High_CTL	0.032015624	-0.142074011
TCGA-49-6742	0.059710543	0.026731925	Low_CTL	0.026731925	-0.275193848
TCGA-49-6743	0.048778282	0.041990366	Low_CTL	0.041990366	0.109233981
TCGA-49-6744	0.010166696	0.043543071	High_CTL	0.010166696	-0.692545467
TCGA-49-6745	0.020089455	0.051031229	High_CTL	0.020089455	-0.442547147
TCGA-49-6761	0.055019507	0.049359446	High_CTL	0.055019507	0.437495864
TCGA-49-6767	0.005760858	0.042395384	High_CTL	0.005760858	-0.803548065
TCGA-49-AAQV	0.03767768	0.028607581	Low_CTL	0.028607581	-0.227937745
TCGA-49-AAR0	0.080480792	0.021230034	High_CTL	0.080480792	1.078978602
TCGA-49-AAR2	0.14708356	0.009480868	Low_CTL	0.009480868	-0.709824511
TCGA-49-AAR3	0.01819535	0.043704103	High_CTL	0.01819535	-0.490268033
TCGA-49-AAR4	0.130697209	0.046035452	High_CTL	0.130697209	2.344152978
TCGA-49-AAR9	0.073048541	0.03598831	Low_CTL	0.03598831	-0.041984437
TCGA-49-AARE	0.122106442	0.02668888	Low_CTL	0.02668888	-0.276278339
TCGA-49-AARN	0.095676142	0.044103913	Low_CTL	0.044103913	0.162483612
TCGA-49-AARO	0.104265902	0.051408392	High_CTL	0.104265902	1.678231061
TCGA-49-AARQ	0.086630724	0.022100853	High_CTL	0.086630724	1.233922689
TCGA-49-AARR	0.074009526	0.013726609	High_CTL	0.074009526	0.915938692
TCGA-4B-A93V	0.083302961	0.029931305	High_CTL	0.083302961	1.150081568
TCGA-50-5044	0.109418347	0.05375593	High_CTL	0.109418347	1.80804402
TCGA-50-5045	0.017963566	0.032487435	High_CTL	0.017963566	-0.4961077
TCGA-50-5049	-0.044025535	0.031718723	High_CTL	-0.044025535	-2.057888222
TCGA-50-5051	0.061913742	0.026469156	Low_CTL	0.026469156	-0.281814157
TCGA-50-5055	0.027533418	0.043287336	High_CTL	0.027533418	-0.25500068
TCGA-50-5066	-0.075179293	0.029136065	High_CTL	-0.075179293	-2.842789627
TCGA-50-5068	-0.074291731	0.024149801	High_CTL	-0.074291731	-2.82042799
TCGA-50-5072	0.092776564	0.048311388	Low_CTL	0.048311388	0.268488572
TCGA-50-5930	0.098125931	0.045823516	High_CTL	0.098125931	1.523537946
TCGA-50-5931	-0.02250222	0.082351282	Low_CTL	0.082351282	1.126104553
TCGA-50-5932	0.048165984	0.017691149	Low_CTL	0.017691149	-0.502971112
TCGA-50-5933	0.025745329	0.080338277	Low_CTL	0.080338277	1.07538801
TCGA-50-5935	-0.032005874	0.028464146	Low_CTL	0.028464146	-0.231551504
TCGA-50-5936	0.06865745	0.050681188	Low_CTL	0.050681188	0.328194354
TCGA-50-5939	0.095483799	0.058168909	Low_CTL	0.058168909	0.516843269
TCGA-50-5941	0.012575348	0.025591484	High_CTL	0.012575348	-0.631860843
TCGA-50-5942	0.029190005	0.018136232	Low_CTL	0.018136232	-0.491757497
TCGA-50-5944	0.005878548	0.052602608	Low_CTL	0.052602608	0.376603441
TCGA-50-5946	0.074949139	0.064478103	Low_CTL	0.064478103	0.675799865

TCGA-50-6590	0.036750469	0.045181894	High_CTL	0.036750469	-0.022782265
TCGA-50-6591	-0.059783191	0.103883653	Low_CTL	0.103883653	1.668600507
TCGA-50-6592	0.082882704	0.027185594	High_CTL	0.082882704	1.139493418
TCGA-50-6593	0.02661713	0.064624537	Low_CTL	0.064624537	0.679489182
TCGA-50-6594	-0.045364995	0.065766779	Low_CTL	0.065766779	0.708267323
TCGA-50-6595	0.088847223	0.099278568	High_CTL	0.088847223	1.28976613
TCGA-50-6597	0.060969807	0.028110837	High_CTL	0.060969807	0.587410332
TCGA-50-6673	0.024780669	0.060173832	Low_CTL	0.060173832	0.567356176
TCGA-50-7109	0.03541961	0.045524445	High_CTL	0.03541961	-0.056312504
TCGA-50-8457	0.033088774	0.037336702	High_CTL	0.033088774	-0.115036605
TCGA-50-8459	0.045939408	0.098370787	Low_CTL	0.098370787	1.529706967
TCGA-50-8460	0.036614612	0.021468196	High_CTL	0.036614612	-0.026205094
TCGA-53-7624	0.076500881	0.049357407	High_CTL	0.076500881	0.978706998
TCGA-53-7626	0.032914622	0.038759342	High_CTL	0.032914622	-0.119424279
TCGA-53-7813	0.012888473	0.03617925	High_CTL	0.012888473	-0.623971812
TCGA-53-A4EZ	0.044167227	0.016466894	Low_CTL	0.016466894	-0.533815519
TCGA-55-1592	-0.037931621	0.030207565	Low_CTL	0.030207565	-0.187627052
TCGA-55-1594	-0.003975423	0.045604832	High_CTL	-0.003975423	-1.048848185
TCGA-55-1596	0.050606777	0.039458045	Low_CTL	0.039458045	0.04543359
TCGA-55-5899	-0.0511977	0.050485591	High_CTL	-0.0511977	-2.238586888
TCGA-55-6543	0.092942144	0.00636084	Low_CTL	0.00636084	-0.788431874
TCGA-55-6642	0.029987127	0.097705747	High_CTL	0.029987127	-0.193180851
TCGA-55-6712	0.080511586	0.038711389	High_CTL	0.080511586	1.07975445
TCGA-55-6968	-0.027574139	0.033581364	High_CTL	-0.027574139	-1.643404558
TCGA-55-6970	0.026725523	0.031419516	High_CTL	0.026725523	-0.275355135
TCGA-55-6971	0.001092709	0.02327257	High_CTL	0.001092709	-0.92115947
TCGA-55-6972	0.004333151	0.017153397	Low_CTL	0.017153397	-0.516519474
TCGA-55-6975	0.087882801	0.09623755	Low_CTL	0.09623755	1.475961255
TCGA-55-6978	0.067858462	0.080959308	High_CTL	0.067858462	0.76096611
TCGA-55-6979	0.032995482	0.029830966	High_CTL	0.032995482	-0.117387038
TCGA-55-6980	0.012465729	0.062381447	Low_CTL	0.062381447	0.6229758
TCGA-55-6981	0.058587178	0.052322548	Low_CTL	0.052322548	0.369547507
TCGA-55-6982	0.043890781	0.048909066	Low_CTL	0.048909066	0.283546731
TCGA-55-6983	0.028009952	0.053982621	High_CTL	0.028009952	-0.242994682
TCGA-55-6984	-0.02112329	0.020812689	Low_CTL	0.020812689	-0.424325662
TCGA-55-6985	0.051002821	0.056871075	High_CTL	0.051002821	0.336297718
TCGA-55-6986	0.10933582	0.043061431	Low_CTL	0.043061431	0.136218874
TCGA-55-6987	-0.027749282	0.027842272	High_CTL	-0.027749282	-1.64781718
TCGA-55-7227	0.063728108	0.04896894	High_CTL	0.063728108	0.656904152
TCGA-55-7281	0.083489051	0.035090945	High_CTL	0.083489051	1.154770002
TCGA-55-7283	0.02704411	0.025505788	High_CTL	0.02704411	-0.267328528
TCGA-55-7570	0.000727861	0.050940222	High_CTL	0.000727861	-0.930351586
TCGA-55-7573	0.036913942	0.028883673	Low_CTL	0.028883673	-0.220981764
TCGA-55-7574	0.098584368	0.057144976	High_CTL	0.098584368	1.535088009

TCGA-55-7576	0.049529565	0.054248362	Low_CTL	0.054248362	0.41806729
TCGA-55-7724	-0.03973861	0.023099059	High_CTL	-0.03973861	-1.949881563
TCGA-55-7725	-0.031800296	0.013527682	High_CTL	-0.031800296	-1.749880206
TCGA-55-7726	0.073997852	0.124985243	Low_CTL	0.124985243	2.200243209
TCGA-55-7727	-0.03862352	0.02785559	Low_CTL	0.02785559	-0.246883746
TCGA-55-7728	0.030178273	0.001979272	High_CTL	0.030178273	-0.188365039
TCGA-55-7815	-0.074853077	0.01868989	High_CTL	-0.074853077	-2.834570785
TCGA-55-7816	-0.014265121	0.046295553	High_CTL	-0.014265121	-1.308091329
TCGA-55-7903	0.006963648	0.044612039	High_CTL	0.006963648	-0.773244449
TCGA-55-7907	0.058657154	0.044027778	High_CTL	0.058657154	0.52914434
TCGA-55-7910	0.077598303	0.040819937	Low_CTL	0.040819937	0.07974569
TCGA-55-7911	0.080924549	0.024818998	High_CTL	0.080924549	1.090158818
TCGA-55-7914	0.02864714	0.062209274	High_CTL	0.02864714	-0.22694109
TCGA-55-7994	0.023252474	0.023697651	High_CTL	0.023252474	-0.362856655
TCGA-55-7995	-0.033296764	0.008433353	High_CTL	-0.033296764	-1.787582881
TCGA-55-8085	0.02215099	0.039119042	High_CTL	0.02215099	-0.390607915
TCGA-55-8087	0.072621304	0.032372924	Low_CTL	0.032372924	-0.13307204
TCGA-55-8089	0.051033346	0.023564111	High_CTL	0.051033346	0.337066776
TCGA-55-8090	0.093899129	0.038053419	Low_CTL	0.038053419	0.010044815
TCGA-55-8091	0.042774737	0.054337625	Low_CTL	0.054337625	0.420316226
TCGA-55-8092	0.078898078	0.037504356	High_CTL	0.078898078	1.039103015
TCGA-55-8094	0.055255812	0.018730871	Low_CTL	0.018730871	-0.476775885
TCGA-55-8096	0.017408986	0.050933745	Low_CTL	0.050933745	0.334557384
TCGA-55-8097	0.125124531	0.032106858	Low_CTL	0.032106858	-0.139775435
TCGA-55-8203	0.01042264	0.032851016	High_CTL	0.01042264	-0.68609709
TCGA-55-8204	0.076300387	0.045175307	High_CTL	0.076300387	0.973655652
TCGA-55-8205	0.010152951	0.029700001	High_CTL	0.010152951	-0.692891756
TCGA-55-8206	-0.026267432	0.013054368	Low_CTL	0.013054368	-0.619792189
TCGA-55-8207	0.024113315	0.033329054	Low_CTL	0.033329054	-0.108982896
TCGA-55-8208	0.004382495	0.023959536	High_CTL	0.004382495	-0.838275148
TCGA-55-8299	0.093430049	0.044311403	High_CTL	0.093430049	1.405227853
TCGA-55-8301	0.007970334	0.035220116	High_CTL	0.007970334	-0.747881557
TCGA-55-8302	0.12394424	0.03615553	Low_CTL	0.03615553	-0.037771414
TCGA-55-8505	0.114459395	0.066569737	Low_CTL	0.066569737	0.728497417
TCGA-55-8506	0.010185992	0.032772307	High_CTL	0.010185992	-0.692059309
TCGA-55-8507	0.068292311	0.041521434	Low_CTL	0.041521434	0.097419511
TCGA-55-8508	0.106720642	0.042816513	High_CTL	0.106720642	1.740076862
TCGA-55-8510	0.02819389	0.033314299	High_CTL	0.02819389	-0.23836045
TCGA-55-8511	0.069157563	0.057356329	High_CTL	0.069157563	0.79369623
TCGA-55-8512	0.021817956	0.008425755	Low_CTL	0.008425755	-0.73640748
TCGA-55-8513	0.007270769	0.007955241	Low_CTL	0.007955241	-0.748261834
TCGA-55-8514	0.126858578	0.030356709	Low_CTL	0.030356709	-0.183869448
TCGA-55-8614	0.110497859	0.100285994	Low_CTL	0.100285994	1.577959522
TCGA-55-8615	0.116421116	0.027366311	Low_CTL	0.027366311	-0.259210856

TCGA-55-8616	0.104331925	0.052001423	Low_CTL	0.052001423	0.361456926
TCGA-55-8619	-0.016937662	0.012078585	High_CTL	-0.016937662	-1.375424512
TCGA-55-8620	0.028251706	0.027853922	High_CTL	0.028251706	-0.236903807
TCGA-55-8621	0.025061441	0.013994208	High_CTL	0.025061441	-0.317280753
TCGA-55-A48X	0.086893197	0.040402407	High_CTL	0.086893197	1.240535535
TCGA-55-A48Y	0.052081674	0.075721056	Low_CTL	0.075721056	0.959059748
TCGA-55-A48Z	0.001984629	0.065009325	High_CTL	0.001984629	-0.898688034
TCGA-55-A490	0.05584372	0.061571698	Low_CTL	0.061571698	0.602574629
TCGA-55-A491	0.109625983	0.039010837	High_CTL	0.109625983	1.813275281
TCGA-55-A492	0.085625387	0.013712701	Low_CTL	0.013712701	-0.603205873
TCGA-55-A493	0.075372235	0.05940734	High_CTL	0.075372235	0.950271381
TCGA-55-A494	0.129398329	0.051076379	Low_CTL	0.051076379	0.338150979
TCGA-55-A4DF	-0.023930158	0.035190319	High_CTL	-0.023930158	-1.551596503
TCGA-55-A4DG	0.028090965	0.041226288	Low_CTL	0.041226288	0.089983474
TCGA-55-A57B	0.076950857	0.070566723	Low_CTL	0.070566723	0.829199207
TCGA-62-8394	0.036293376	0.03881456	Low_CTL	0.03881456	0.029221337
TCGA-62-8395	0.1166454	0.022852728	Low_CTL	0.022852728	-0.37292802
TCGA-62-8397	0.025883345	0.02341399	High_CTL	0.025883345	-0.296573332
TCGA-62-8398	0.090940453	0.041707643	Low_CTL	0.041707643	0.102110948
TCGA-62-8399	0.102731548	0.078106528	Low_CTL	0.078106528	1.019160367
TCGA-62-8402	0.078541821	0.027845746	High_CTL	0.078541821	1.030127317
TCGA-62-A46O	0.073372449	0.034162901	Low_CTL	0.034162901	-0.087974586
TCGA-62-A46P	0.104236463	0.014487577	Low_CTL	0.014487577	-0.583683303
TCGA-62-A46R	-0.01752566	0.037378698	Low_CTL	0.037378698	-0.006954391
TCGA-62-A46S	0.037703251	0.005644661	Low_CTL	0.005644661	-0.806475589
TCGA-62-A46V	0.047633548	0.045875927	Low_CTL	0.045875927	0.207128501
TCGA-62-A46Y	0.008629177	0.01479409	High_CTL	0.008629177	-0.731282385
TCGA-62-A470	0.067047764	0.019211695	Low_CTL	0.019211695	-0.464661792
TCGA-62-A471	0.137418927	0.023939476	High_CTL	0.137418927	2.513502863
TCGA-62-A472	0.031735043	0.039364588	High_CTL	0.031735043	-0.1491431
TCGA-64-1676	-0.042418397	0.020673057	High_CTL	-0.042418397	-2.017397292
TCGA-64-1677	0.030580525	0.044858286	High_CTL	0.030580525	-0.178230523
TCGA-64-1678	-0.015016939	0.045600155	Low_CTL	0.045600155	0.200180589
TCGA-64-1679	0.071766431	0.088222399	Low_CTL	0.088222399	1.274024043
TCGA-64-1680	0.037283713	0.024327021	Low_CTL	0.024327021	-0.335784051
TCGA-64-1681	0.012843001	0.034173324	Low_CTL	0.034173324	-0.087711986
TCGA-64-5774	0.05723548	0.020517656	Low_CTL	0.020517656	-0.431758858
TCGA-64-5775	-0.048450334	0.073870921	Low_CTL	0.073870921	0.912446617
TCGA-64-5778	-0.011736397	0.020187283	High_CTL	-0.011736397	-1.244381561
TCGA-64-5779	0.065701147	0.091271523	High_CTL	0.065701147	0.70661376
TCGA-64-5781	0.037202083	0.044142457	High_CTL	0.037202083	-0.011404093
TCGA-64-5815	0.0717896	0.086536573	Low_CTL	0.086536573	1.231550599
TCGA-67-3770	-0.032140927	0.01905865	High_CTL	-0.032140927	-1.758462212
TCGA-67-3771	0.022291443	0.024642489	High_CTL	0.022291443	-0.387069296

TCGA-67-3772	-0.0263541	0.043882713	High_CTL	-0.0263541	-1.612666366
TCGA-67-3773	-0.06286027	0.01432604	High_CTL	-0.06286027	-2.532418765
TCGA-67-3774	0.049604766	0.024099679	Low_CTL	0.024099679	-0.34151179
TCGA-67-6215	0.054904931	0.01413818	Low_CTL	0.01413818	-0.592486172
TCGA-67-6216	0.086004166	0.022183281	Low_CTL	0.022183281	-0.38979436
TCGA-67-6217	-0.004739466	0.01819487	High_CTL	-0.004739466	-1.068097827
TCGA-69-7760	0.014913324	0.053172231	Low_CTL	0.053172231	0.390954789
TCGA-69-7761	0.007442033	0.027419265	High_CTL	0.007442033	-0.761191825
TCGA-69-7763	0.118901296	0.048640793	Low_CTL	0.048640793	0.276787755
TCGA-69-7764	0.064111557	0.019039497	Low_CTL	0.019039497	-0.469000236
TCGA-69-7765	0.009781276	0.064521594	High_CTL	0.009781276	-0.702255914
TCGA-69-7973	0.047880655	0.060356035	Low_CTL	0.060356035	0.571946674
TCGA-69-7974	0.101039151	0.029176651	High_CTL	0.101039151	1.596934892
TCGA-69-7978	0.012681925	0.040106264	High_CTL	0.012681925	-0.629175683
TCGA-69-7979	0.084192961	0.052021978	Low_CTL	0.052021978	0.361974803
TCGA-69-7980	0.099500475	0.04284132	High_CTL	0.099500475	1.558168824
TCGA-69-8253	0.117257941	0.022042007	Low_CTL	0.022042007	-0.393353695
TCGA-69-8254	0.056071266	0.018303818	Low_CTL	0.018303818	-0.487535249
TCGA-69-8255	-0.002861849	0.014084774	High_CTL	-0.002861849	-1.020792331
TCGA-69-8453	-0.007112422	0.007086638	High_CTL	-0.007112422	-1.127883126
TCGA-69-A59K	-0.019895726	0.04039178	High_CTL	-0.019895726	-1.449951262
TCGA-71-6725	0.094884135	0.028501196	Low_CTL	0.028501196	-0.230618058
TCGA-71-8520	0.018301416	0.080720124	Low_CTL	0.080720124	1.085008441
TCGA-73-4658	0.042993731	0.060129343	High_CTL	0.042993731	0.134513206
TCGA-73-4659	-0.004188331	0.023498859	Low_CTL	0.023498859	-0.356649125
TCGA-73-4662	-0.021483824	0.031172602	Low_CTL	0.031172602	-0.163313479
TCGA-73-4666	-0.007368802	0.027843885	High_CTL	-0.007368802	-1.134342471
TCGA-73-4668	0.074509214	0.071198692	Low_CTL	0.071198692	0.845121326
TCGA-73-4670	0.107951237	0.052592596	Low_CTL	0.052592596	0.376351216
TCGA-73-4675	0.070402982	0.028736952	Low_CTL	0.028736952	-0.224678319
TCGA-73-4676	-0.031783043	0.043808194	Low_CTL	0.043808194	0.155033131
TCGA-73-4677	0.039319057	0.015360066	Low_CTL	0.015360066	-0.561701435
TCGA-73-7498	0.069341041	0.025994831	Low_CTL	0.025994831	-0.293764514
TCGA-73-7499	-0.032967893	0.022436441	High_CTL	-0.032967893	-1.779297153
TCGA-73-A9RS	0.104794781	0.042670276	Low_CTL	0.042670276	0.126363939
TCGA-78-7143	0.027839032	0.03066361	Low_CTL	0.03066361	-0.176137257
TCGA-78-7145	0.023713382	0.054286985	Low_CTL	0.054286985	0.419040396
TCGA-78-7146	0.062806075	0.047843569	Low_CTL	0.047843569	0.256702137
TCGA-78-7147	0.033159196	0.039700096	Low_CTL	0.039700096	0.051531917
TCGA-78-7148	0.070114656	0.028358746	High_CTL	0.070114656	0.817809647
TCGA-78-7149	0.104276538	0.023201418	Low_CTL	0.023201418	-0.36414298
TCGA-78-7150	0.05933743	0.070013386	Low_CTL	0.070013386	0.815258195
TCGA-78-7152	0.086036278	0.031268768	High_CTL	0.086036278	1.218945952
TCGA-78-7153	0.073644808	0.034274065	Low_CTL	0.034274065	-0.085173883

TCGA-78-7154	0.007619436	0.03274957	Low_CTL	0.03274957	-0.123582658
TCGA-78-7155	-0.005831871	0.089108141	Low_CTL	0.089108141	1.296339805
TCGA-78-7156	0.031526226	0.018264474	Low_CTL	0.018264474	-0.488526511
TCGA-78-7158	-0.024398849	0.047197264	Low_CTL	0.047197264	0.240418858
TCGA-78-7159	0.027642122	0.031496993	High_CTL	0.027642122	-0.252261932
TCGA-78-7160	0.014354264	0.03544674	High_CTL	0.014354264	-0.587042055
TCGA-78-7161	0.078961949	0.010119174	Low_CTL	0.010119174	-0.693742745
TCGA-78-7162	-0.002324318	0.013891084	High_CTL	-0.002324318	-1.007249523
TCGA-78-7163	0.058151624	0.009244152	Low_CTL	0.009244152	-0.715788448
TCGA-78-7166	0.132540325	0.031913779	Low_CTL	0.031913779	-0.144639939
TCGA-78-7167	0.073858871	0.020459718	Low_CTL	0.020459718	-0.433218571
TCGA-78-7220	0.030363497	0.041526859	High_CTL	0.030363497	-0.183698426
TCGA-78-7535	0.089716964	0.030585404	Low_CTL	0.030585404	-0.178107615
TCGA-78-7536	0.029026862	0.016314896	Low_CTL	0.016314896	-0.537645036
TCGA-78-7537	0.082402835	0.022806945	Low_CTL	0.022806945	-0.374081503
TCGA-78-7539	-0.003902281	0.025227527	High_CTL	-0.003902281	-1.047005408
TCGA-78-7540	0.035696862	0.011954795	Low_CTL	0.011954795	-0.647495308
TCGA-78-7542	0.058052447	0.040258121	Low_CTL	0.040258121	0.065591041
TCGA-78-7633	-0.005312636	0.008138851	Low_CTL	0.008138851	-0.743635883
TCGA-78-8640	0.031710931	0.024271963	High_CTL	0.031710931	-0.1497506
TCGA-78-8648	0.019448434	0.059045532	High_CTL	0.019448434	-0.45869729
TCGA-78-8655	0.061350948	0.018014361	High_CTL	0.061350948	0.597012945
TCGA-78-8660	0.099531918	0.025641763	High_CTL	0.099531918	1.558960996
TCGA-78-8662	0.090026492	0.053178847	Low_CTL	0.053178847	0.39112146
TCGA-83-5908	0.036653553	0.048721998	High_CTL	0.036653553	-0.025224012
TCGA-86-6562	0.030963763	0.055241273	Low_CTL	0.055241273	0.443083141
TCGA-86-6851	0.028017949	0.020198696	High_CTL	0.028017949	-0.242793183
TCGA-86-7701	-0.014152053	0.062535525	High_CTL	-0.014152053	-1.305242647
TCGA-86-7711	-0.007227407	-0.009749669	High_CTL	-0.007227407	-1.130780087
TCGA-86-7713	0.083802927	0.038332659	Low_CTL	0.038332659	0.01708011
TCGA-86-7714	0.040095808	0.026952363	Low_CTL	0.026952363	-0.269640033
TCGA-86-7953	-0.005277367	0.045068856	High_CTL	-0.005277367	-1.081649949
TCGA-86-7954	0.007075666	0.023722781	High_CTL	0.007075666	-0.770422213
TCGA-86-7955	0.107378663	0.037371752	Low_CTL	0.037371752	-0.007129393
TCGA-86-8054	0.042264219	0.053091634	Low_CTL	0.053091634	0.388924178
TCGA-86-8055	0.032365803	0.104285219	Low_CTL	0.104285219	1.678717736
TCGA-86-8056	0.057534348	0.032093727	Low_CTL	0.032093727	-0.140106254
TCGA-86-8073	0.044061139	0.038651905	Low_CTL	0.038651905	0.025123338
TCGA-86-8074	0.006426846	0.086739207	Low_CTL	0.086739207	1.236655858
TCGA-86-8075	0.023840941	0.089521309	Low_CTL	0.089521309	1.306749348
TCGA-86-8076	0.05157483	0.038884595	High_CTL	0.05157483	0.350709169
TCGA-86-8278	0.100596245	0.054301172	Low_CTL	0.054301172	0.419397824
TCGA-86-8279	0.100232311	0.073155571	Low_CTL	0.073155571	0.894423784
TCGA-86-8280	-0.000750091	0.041448911	High_CTL	-0.000750091	-0.967587778

TCGA-86-8358	-0.023685659	0.038846653	High_CTL	-0.023685659	-1.545436501
TCGA-86-8359	0.102455928	0.01634138	High_CTL	0.102455928	1.632629801
TCGA-86-8585	0.062448828	0.031412109	High_CTL	0.062448828	0.62467343
TCGA-86-8668	0.075995487	0.043173179	Low_CTL	0.043173179	0.139034299
TCGA-86-8669	0.05028612	0.016634352	Low_CTL	0.016634352	-0.529596501
TCGA-86-8671	0.017253497	0.023799238	High_CTL	0.017253497	-0.513997507
TCGA-86-8672	0.109486727	0.054122706	High_CTL	0.109486727	1.809766809
TCGA-86-8673	0.066597739	0.048088137	High_CTL	0.066597739	0.729202903
TCGA-86-8674	0.096099345	0.019368152	Low_CTL	0.019368152	-0.460719962
TCGA-86-A456	0.048767446	0.019612852	Low_CTL	0.019612852	-0.454554888
TCGA-86-A4D0	0.033355929	0.050196972	High_CTL	0.033355929	-0.108305782
TCGA-86-A4JF	0.10107846	0.018647054	High_CTL	0.10107846	1.597925257
TCGA-86-A4P7	0.063578594	0.028052318	High_CTL	0.063578594	0.653137233
TCGA-86-A4P8	-0.012133807	0.008095356	High_CTL	-0.012133807	-1.254394079
TCGA-91-6828	0.018461124	0.01883311	High_CTL	0.018461124	-0.483572022
TCGA-91-6829	0.020097892	0.126641296	Low_CTL	0.126641296	2.241966511
TCGA-91-6830	0.053097518	0.058158391	High_CTL	0.053097518	0.389072436
TCGA-91-6831	0.022050654	0.085159324	High_CTL	0.022050654	-0.39313582
TCGA-91-6835	-0.031870665	0.025154147	High_CTL	-0.031870665	-1.751653102
TCGA-91-6836	-0.022535649	0.053581038	Low_CTL	0.053581038	0.401254444
TCGA-91-6840	0.048269121	0.038131914	High_CTL	0.048269121	0.267423697
TCGA-91-6847	0.036082987	0.048244704	Low_CTL	0.048244704	0.266808525
TCGA-91-6848	-0.007120328	0.101424255	High_CTL	-0.007120328	-1.128082303
TCGA-91-6849	0.062238177	0.019818495	High_CTL	0.062238177	0.6193662
TCGA-91-7771	0.047695272	0.029776099	High_CTL	0.047695272	0.252965888
TCGA-91-8496	-0.052836507	-0.000198731	High_CTL	-0.052836507	-2.279875693
TCGA-91-8497	0.035212539	0.022024105	Low_CTL	0.022024105	-0.393804723
TCGA-91-8499	-0.084965496	0.057621233	High_CTL	-0.084965496	-3.0893475
TCGA-91-A4BC	0.066816649	0.084374736	High_CTL	0.066816649	0.734718206
TCGA-91-A4BD	0.008106142	-0.000203667	Low_CTL	-0.000203667	-0.953820919
TCGA-93-7347	0.003281489	0.02804136	High_CTL	0.003281489	-0.866014373
TCGA-93-7348	0.067426316	0.063842908	Low_CTL	0.063842908	0.659796479
TCGA-93-8067	0.115985236	0.032428482	Low_CTL	0.032428482	-0.131672288
TCGA-93-A4JN	0.11851189	0.092388398	Low_CTL	0.092388398	1.378984029
TCGA-93-A4JO	0.136256099	0.040911458	High_CTL	0.136256099	2.48420608
TCGA-93-A4JP	0.013195396	0.01848207	Low_CTL	0.01848207	-0.483044297
TCGA-93-A4JQ	0.088429984	0.089175188	High_CTL	0.088429984	1.279254016
TCGA-95-7039	0.084815732	0.059861923	Low_CTL	0.059861923	0.559497819
TCGA-95-7043	-0.022926931	0.012286801	Low_CTL	0.012286801	-0.639130617
TCGA-95-7562	-0.048475532	0.042297798	Low_CTL	0.042297798	0.116979576
TCGA-95-7567	0.046927099	0.049251396	High_CTL	0.046927099	0.233612206
TCGA-95-7944	0.076449696	0.058054355	High_CTL	0.076449696	0.977417421
TCGA-95-7947	-0.012358582	0.031735633	Low_CTL	0.031735633	-0.149128236
TCGA-95-7948	0.068000472	0.05512244	Low_CTL	0.05512244	0.44008921

TCGA-95-8039	0.051020773	0.031952467	Low_CTL	0.031952467	-0.143665214
TCGA-95-8494	0.042340359	0.043752208	Low_CTL	0.043752208	0.15362261
TCGA-95-A4VK	0.144405815	0.04416001	Low_CTL	0.04416001	0.163896941
TCGA-95-A4VN	0.105828958	0.034036279	High_CTL	0.105828958	1.717611387
TCGA-95-A4VP	0.14672854	0.03236159	High_CTL	0.14672854	2.748053322
TCGA-97-7546	0.003926499	0.035024532	Low_CTL	0.035024532	-0.066266266
TCGA-97-7547	0.055866136	0.009946351	Low_CTL	0.009946351	-0.698096936
TCGA-97-7552	-0.02985102	0.001919537	High_CTL	-0.02985102	-1.700769304
TCGA-97-7553	-0.035966704	0.007497501	High_CTL	-0.035966704	-1.854850498
TCGA-97-7554	-0.006832918	0.074817517	Low_CTL	0.074817517	0.936295582
TCGA-97-7937	0.066977534	0.064210401	Low_CTL	0.064210401	0.669055256
TCGA-97-7938	0.041846978	0.046051534	Low_CTL	0.046051534	0.211552836
TCGA-97-7941	0.074199776	0.041056283	Low_CTL	0.041056283	0.085700304
TCGA-97-8171	-0.007360623	0.032569248	Low_CTL	0.032569248	-0.128125785
TCGA-97-8172	0.034363244	0.037258505	High_CTL	0.034363244	-0.08292706
TCGA-97-8174	0.076758181	0.010275679	Low_CTL	0.010275679	-0.689799708
TCGA-97-8175	0.083209603	0.025338925	Low_CTL	0.025338925	-0.310289686
TCGA-97-8176	0.077971312	0.040687802	Low_CTL	0.040687802	0.076416615
TCGA-97-8177	-0.011753647	0.023478135	High_CTL	-0.011753647	-1.244816174
TCGA-97-8179	-0.004952781	0.031885927	Low_CTL	0.031885927	-0.14534167
TCGA-97-8547	0.061476248	0.084023894	Low_CTL	0.084023894	1.168245079
TCGA-97-8552	0.019866597	0.007763637	Low_CTL	0.007763637	-0.753089171
TCGA-97-A4LX	0.006719089	0.043942057	High_CTL	0.006719089	-0.779405984
TCGA-97-A4M0	0.038989315	0.013812038	High_CTL	0.038989315	0.033624191
TCGA-97-A4M1	-0.005922194	0.020975199	Low_CTL	0.020975199	-0.420231314
TCGA-97-A4M2	0.011479374	0.007118103	High_CTL	0.011479374	-0.659473283
TCGA-97-A4M3	0.109076128	0.015407835	Low_CTL	0.015407835	-0.560497927
TCGA-97-A4M5	0.046856909	0.037493798	High_CTL	0.046856909	0.231843812
TCGA-97-A4M6	0.025132431	0.028497643	High_CTL	0.025132431	-0.315492206
TCGA-97-A4M7	0.067835535	0.041297097	Low_CTL	0.041297097	0.091767457
TCGA-99-7458	-0.044056784	0.048102936	High_CTL	-0.044056784	-2.058675532
TCGA-99-8025	0.125840201	0.051642953	Low_CTL	0.051642953	0.352425481
TCGA-99-8028	0.01934612	0.040849663	High_CTL	0.01934612	-0.461275039
TCGA-99-8032	0.070573662	0.050046685	Low_CTL	0.050046685	0.312208417
TCGA-99-8033	0.133038377	0.048900122	High_CTL	0.133038377	2.403137373
TCGA-99-AA5R	0.029502421	0.023583855	High_CTL	0.029502421	-0.205392745
TCGA-J2-8192	0.015403971	0.086915957	Low_CTL	0.086915957	1.241108963
TCGA-J2-8194	0.093248223	0.045383836	Low_CTL	0.045383836	0.19473055
TCGA-J2-A4AD	0.070753738	0.061220541	Low_CTL	0.061220541	0.593727436
TCGA-J2-A4AE	0.0839224	0.007117307	Low_CTL	0.007117307	-0.7693731
TCGA-J2-A4AG	0.054375466	0.044970993	High_CTL	0.054375466	0.42126961
TCGA-L4-A4E5	0.103892022	0.061526287	Low_CTL	0.061526287	0.601430524
TCGA-L4-A4E6	-0.023993197	0.004962143	Low_CTL	0.004962143	-0.823671237
TCGA-L9-A443	0.079924309	0.061100356	Low_CTL	0.061100356	0.590699422

TCGA-L9-A444	0.058673269	0.027829519	High_CTL	0.058673269	0.52955035
TCGA-L9-A50W	0.109642928	0.056534607	High_CTL	0.109642928	1.813702214
TCGA-L9-A5IP	0.116536546	0.021610707	High_CTL	0.116536546	1.987383039
TCGA-L9-A743	0.073756104	0.044341192	High_CTL	0.073756104	0.909553879
TCGA-L9-A7SV	0.082168838	0.061173806	Low_CTL	0.061173806	0.592549974
TCGA-L9-A8F4	0.096249851	0.047694791	High_CTL	0.096249851	1.476271179
TCGA-MN-A4N1	0.095506298	0.050527055	Low_CTL	0.050527055	0.324311072
TCGA-MN-A4N4	0.078948637	0.075729972	Low_CTL	0.075729972	0.959284375
TCGA-MN-A4N5	0.112690999	0.036165104	High_CTL	0.112690999	1.890496632
TCGA-MP-A4SV	0.068457103	0.054228382	High_CTL	0.068457103	0.776048527
TCGA-MP-A4SW	0.095038632	0.018097859	Low_CTL	0.018097859	-0.492724284
TCGA-MP-A4SY	0.093904327	0.070072513	Low_CTL	0.070072513	0.816747877
TCGA-MP-A4T4	0.027717895	0.043657608	High_CTL	0.027717895	-0.250352882
TCGA-MP-A4T6	0.101048286	0.024898165	Low_CTL	0.024898165	-0.321394386
TCGA-MP-A4T7	0.141198763	0.028276587	Low_CTL	0.028276587	-0.236276941
TCGA-MP-A4T8	0.108794787	0.080630618	Low_CTL	0.080630618	1.082753382
TCGA-MP-A4T9	0.055640231	0.04620226	Low_CTL	0.04620226	0.215350281
TCGA-MP-A4TA	0.111225342	0.039611696	High_CTL	0.111225342	1.853570241
TCGA-MP-A4TC	0.098025641	0.080404613	High_CTL	0.098025641	1.521011214
TCGA-MP-A4TD	0.099402647	0.074248805	Low_CTL	0.074248805	0.921967191
TCGA-MP-A4TE	0.068173856	0.026449779	Low_CTL	0.026449779	-0.282302355
TCGA-MP-A4TF	0.088998122	0.047353858	High_CTL	0.088998122	1.293567948
TCGA-MP-A4TH	0.093717682	0.005808632	High_CTL	0.093717682	1.4124746
TCGA-MP-A4TI	0.103039999	0.047830529	High_CTL	0.103039999	1.647345122
TCGA-MP-A4TJ	0.058469283	0.03911323	High_CTL	0.058469283	0.524411039
TCGA-MP-A4TK	0.091392636	0.091118137	Low_CTL	0.091118137	1.346980539
TCGA-MP-A5C7	0.055124298	0.009088197	Low_CTL	0.009088197	-0.719717627
TCGA-NJ-A4YF	0.109348965	0.04450355	Low_CTL	0.04450355	0.172552234
TCGA-NJ-A4YG	0.070273396	0.026966345	Low_CTL	0.026966345	-0.269287751
TCGA-NJ-A4YI	-0.000788572	0.037867187	Low_CTL	0.037867187	0.005352826
TCGA-NJ-A4YP	0.119945251	0.087415417	High_CTL	0.119945251	2.073263433
TCGA-NJ-A4YQ	-0.013962461	-0.002740214	High_CTL	-0.013962461	-1.300465999
TCGA-NJ-A55A	0.072210647	0.017020609	High_CTL	0.072210647	0.870616963
TCGA-NJ-A55O	0.075382596	0.011064067	Low_CTL	0.011064067	-0.6699367
TCGA-NJ-A55R	0.056472349	0.03461132	Low_CTL	0.03461132	-0.076676919
TCGA-NJ-A7XG	0.144021576	0.009714345	Low_CTL	0.009714345	-0.703942184
TCGA-O1-A52J	-0.023288163	0.013634258	Low_CTL	0.013634258	-0.605182195
TCGA-S2-AA1A	-0.00682305	0.049922658	High_CTL	-0.00682305	-1.120592542

[a] When the expression level of CTL markers (*CD8A*, *CD8B*, *GZMA*, *GZMB*, *PRF1*) is higher than the average expression level, we refer to it as a CTL high-expressing tumor; conversely, it is a CTL low-expressing tumor.

[b] The TIDE score for samples with high CTL expression utilizes the T-cell dysfunction score, while the TIDE score for samples with low CTL expression employs the T-cell exclusion score.

Supplementary Table 3. The auc for predicting 1, 3, and 5 year survival on the 20 train sets

		auc_1year_train	auc_3year_train	auc_5year_train
gene+micro	1	0.718788359	0.704168331	0.629307083
	2	0.672412739	0.701871938	0.705626555
	3	0.66911916	0.701930294	0.741617224
	4	0.653736834	0.68568504	0.741196831
	5	0.655122153	0.679617337	0.706503495
	6	0.751440832	0.697059101	0.739351137
	7	0.712025494	0.708272695	0.672371301
	8	0.701026735	0.719265858	0.720587815
	9	0.71005206	0.703482142	0.723798497
	10	0.782448082	0.721423483	0.668060876
	11	0.703239705	0.703478394	0.671727009
	12	0.702178815	0.690588721	0.709272917
	13	0.739119605	0.715701213	0.723924665
	14	0.705405508	0.691719535	0.709380048
	15	0.706961083	0.718905859	0.708818552
	16	0.727041741	0.70507398	0.764553581
	17	0.737084106	0.706319516	0.686646593
	18	0.722434784	0.745322551	0.787786052
	19	0.711063035	0.747581809	0.698741946
	20	0.691313982	0.731780437	0.690898458
gene	1	0.702835289	0.700467367	0.637495177
	2	0.669709009	0.685411816	0.69423241
	3	0.65632205	0.698938737	0.715460233
	4	0.656246406	0.684843016	0.730552815
	5	0.65000124	0.685059089	0.695094683
	6	0.759474127	0.703800835	0.732534235
	7	0.716024025	0.708623383	0.657351141
	8	0.707791609	0.714568594	0.7135043
	9	0.696720648	0.684948286	0.718937205
	10	0.768998381	0.723747996	0.66352463
	11	0.694003061	0.698240071	0.665118876
	12	0.701253228	0.68405	0.714364808
	13	0.733242893	0.702550403	0.72603058
	14	0.689283326	0.691409026	0.688535803
	15	0.701493908	0.720353531	0.710707636
	16	0.704431004	0.688058097	0.757548577
	17	0.737918937	0.713188489	0.675718283
	18	0.719886766	0.74631608	0.779014468
	19	0.697882067	0.739165104	0.687312004

	20	0.689044511	0.730928061	0.697582965
micro	1	0.658693336	0.671302298	0.693648486
	2	0.646036691	0.633937238	0.698579185
	3	0.654207477	0.722905001	0.758649985
	4	0.682441663	0.706516497	0.724463879
	5	0.619660127	0.661012162	0.729175009
	6	0.721508163	0.690017554	0.751502155
	7	0.693327637	0.693828675	0.731049128
	8	0.663375083	0.692195564	0.739160419
	9	0.681730384	0.639045211	0.755091843
	10	0.701361255	0.733402268	0.784602113
	11	0.705189044	0.691231082	0.782173082
	12	0.699304247	0.707523407	0.724672889
	13	0.703238354	0.687836504	0.732193803
	14	0.723100601	0.668218507	0.692129742
	15	0.696962443	0.669943399	0.723724252
	16	0.695348856	0.737450205	0.777376778
	17	0.73780864	0.666887164	0.726637492
	18	0.732615462	0.736928881	0.691708183
	19	0.656883617	0.658323819	0.658030241
	20	0.672808243	0.682030535	0.704388698

Supplementary Table 4. The auc for predicting 1, 3, and 5 year survival on the 20 test sets

		auc_1year_test	auc_3year_test	auc_5year_test
gene+micro	1	0.682599778	0.705039587	0.768683256
	2	0.772575188	0.703282879	0.66265974
	3	0.775392358	0.683348418	0.646782543
	4	0.768150494	0.672826163	0.617482165
	5	0.823595963	0.740544376	0.691081245
	6	0.608542912	0.661736832	0.540367006
	7	0.756396915	0.697639869	0.738153625
	8	0.689430926	0.645537093	0.583289863
	9	0.708286019	0.710506939	0.611828726
	10	0.511613363	0.640849751	0.780985742
	11	0.752598376	0.701403519	0.711950283
	12	0.704919302	0.734749221	0.671086643
	13	0.655473556	0.660693903	0.611430695
	14	0.683344277	0.678154342	0.626416191
	15	0.721131645	0.64876566	0.64806837
	16	0.666934497	0.704505229	0.552023698
	17	0.651764928	0.654585388	0.676730432
	18	0.658995594	0.575036797	0.456201764
	19	0.716806938	0.592712887	0.646166932
	20	0.748714799	0.640354932	0.669123537
gene	1	0.710665282	0.707922052	0.758608831
	2	0.757523137	0.711990137	0.666242088
	3	0.765092787	0.693524475	0.676270494
	4	0.753898685	0.692824539	0.648149855
	5	0.793573288	0.718490972	0.662570988
	6	0.579401084	0.684192698	0.57118655
	7	0.732514931	0.66928598	0.739325358
	8	0.662647594	0.633632583	0.557991518
	9	0.723267775	0.715088816	0.634464193
	10	0.486320528	0.620631183	0.795070353
	11	0.724101294	0.665973422	0.706570048
	12	0.693109783	0.727679375	0.674198456
	13	0.640740505	0.681371133	0.621012523
	14	0.74354081	0.695093714	0.724148477
	15	0.724703649	0.656924562	0.6808176
	16	0.718919469	0.739773473	0.576888151
	17	0.627785794	0.637484007	0.719375348
	18	0.648993961	0.568408802	0.459007291
	19	0.714857131	0.588813342	0.637724116

	20	0.743942511	0.652697411	0.6775956
	1	0.580845526	0.619020762	0.601059846
	2	0.589960437	0.586354621	0.677468082
	3	0.54184678	0.410944304	0.427998806
	4	0.576599633	0.489974885	0.479453869
	5	0.602919652	0.62900153	0.646121083
	6	0.44200574	0.44490063	0.462119167
	7	0.484795972	0.526601814	0.692200492
	8	0.449001544	0.482356583	0.489062221
	9	0.507892904	0.55669666	0.609999435
	10	0.508584662	0.437649843	0.573610795
micro	11	0.556817011	0.560331283	0.433527992
	12	0.512113097	0.519645575	0.523122061
	13	0.473403615	0.568488556	0.595926969
	14	0.593651988	0.584832989	0.648011513
	15	0.479237413	0.588854983	0.607726477
	16	0.501502119	0.53485457	0.403883092
	17	0.489982016	0.574381328	0.525499726
	18	0.490135847	0.435693325	0.509916112
	19	0.602444381	0.559445071	0.658564358
	20	0.667035251	0.523863156	0.55242753

Supplementary Table 5. The C index for Cox models

		c.index	lower	upper
gene+micro	1	0.728323977	0.60714585	0.823019567
	2	0.722284382	0.602600381	0.816877039
	3	0.715309538	0.594549233	0.811505401
	4	0.726542998	0.603983883	0.822330009
	5	0.705231931	0.586990012	0.801093013
	6	0.768836733	0.652437312	0.854922007
	7	0.74725369	0.634240014	0.834462059
	8	0.74686941	0.628086394	0.837528612
	9	0.749516721	0.629242046	0.840654555
	10	0.755478109	0.642146131	0.841762489
	11	0.736574158	0.619865995	0.827426431
	12	0.729247806	0.609543991	0.82291505
	13	0.744249832	0.62388777	0.836206418
	14	0.731959245	0.612667782	0.825004925
	15	0.727910556	0.610387503	0.820413765
	16	0.751821951	0.63811243	0.838826882
	17	0.754434319	0.639783646	0.841626786
	18	0.754066986	0.637853609	0.842212575
	19	0.740109376	0.617945397	0.833721825
	20	0.740083457	0.619651036	0.832679464
gene	1	0.677702605	0.554378403	0.780415675
	2	0.675524476	0.553952207	0.777283633
	3	0.657645339	0.534967367	0.762337717
	4	0.658166512	0.533143406	0.76449802
	5	0.652508091	0.532691892	0.755693063
	6	0.718962466	0.599172762	0.814061869
	7	0.687907655	0.57180259	0.784401223
	8	0.695132285	0.573586891	0.794447853
	9	0.689058573	0.56562985	0.79041034
	10	0.712524504	0.596539235	0.806009696
	11	0.672778879	0.553248839	0.773425405
	12	0.680540566	0.55870468	0.781871752
	13	0.709845058	0.587527367	0.807758439
	14	0.669985044	0.548112738	0.772623839
	15	0.691064639	0.571817779	0.789336571
	16	0.683875191	0.566617296	0.781633946
	17	0.716203601	0.599163516	0.809911287
	18	0.702118934	0.582892726	0.799016262
	19	0.694979554	0.570524735	0.796249253
	20	0.688234448	0.565259193	0.789384908
micro	1	0.638501032	0.514493081	0.746444709

2	0.63958042	0.517367951	0.746037123
3	0.661935971	0.5393381	0.766058733
4	0.673926876	0.549237193	0.778062454
5	0.627337289	0.507276873	0.73351139
6	0.682825743	0.561536994	0.783498981
7	0.661517336	0.544665291	0.761512182
8	0.670181715	0.547862504	0.773113516
9	0.654262517	0.529980593	0.760530728
10	0.688346765	0.571346834	0.785406328
11	0.662416652	0.542640904	0.764441403
12	0.652104547	0.52963582	0.757298165
13	0.669810413	0.546103622	0.773768795
14	0.662787437	0.540754359	0.766401401
15	0.648379504	0.528073254	0.752396767
16	0.686624482	0.569458944	0.783999329
17	0.678773061	0.560230514	0.778022662
18	0.685805423	0.565962901	0.785121283
19	0.629009213	0.503300338	0.739377537
20	0.652837067	0.529009737	0.758943742

Supplementary Table 6. The association of combined data of tme-related genes and microbial biomarkers and LUAD mortality

	coef[a]	HR	lower CI of HR	upper CI of HR
GCSAML	-0.43656995	0.646249291	0.480898445	0.868453934
APOC4-APOC2	-0.240639379	0.786125069	0.597475059	1.034340454
CHRDL1	0.160431122	1.174016907	0.944928425	1.458645608
GPR1	0.158886872	1.17220533	0.998829162	1.375676029
CPS1	0.139997847	1.150271322	0.959014296	1.379670897
IKZF3	-0.303447037	0.738268989	0.58509767	0.931538662
CD70	0.264083529	1.302236966	1.090633586	1.554895373
KLRC2	0.299059321	1.348589621	1.09908074	1.654741002
g__Terrabacter	-0.203150025	0.816155789	0.627177871	1.062075532
g__Effusibacillus	0.1464587	1.157727116	0.954896306	1.403641492
g__Marinithermus	0.19592096	1.216430755	0.962392043	1.537527031
g__Renibacterium	0.276733409	1.31881474	1.09861739	1.583146539
g__Achromobacter	-0.620625975	0.537607803	0.320879747	0.900717955
g__Herbaspirillum	0.249349466	1.283190386	1.035058737	1.590805919
g__Chlamydia	0.193068906	1.212966371	0.948524708	1.551132412
g__Lactobacillus	-4.336280748	0.013085105	0.000153347	1.116553526
g__Aquimarina	-0.205621441	0.814141219	0.632649302	1.047698815
g__Klebsiella	0.619343521	1.857708095	1.146284564	3.01066548
g__Streptococcus	0.227338631	1.255254864	1.003992776	1.569398516

[a] The multivariable Cox regression model coefficient.

Abbreviations: HR, hazard ratiol; CI, confidence interva.
